# Downregulation of MEIS1 mediated by ELFN1-AS1/EZH2/DNMT3a axis promotes tumorigenesis and oxaliplatin resistance in colorectal cancer

**DOI:** 10.1038/s41392-022-00902-6

**Published:** 2022-03-30

**Authors:** Yimin Li, Yaqi Gan, Jiaxin Liu, Juanni Li, Zhengwei Zhou, Ruotong Tian, Ruizheng Sun, Jiaqi Liu, Qing Xiao, Yuanyuan Li, Pengyan Lu, Yulong Peng, Yuqian Peng, Guang Shu, Gang Yin

**Affiliations:** 1 Department of Pathology, Xiangya Hospital, School of Basic Medical Sciences, Central South University, Changsha, China; 2 School of Basic Medical Sciences, Central South University, Changsha, Hunan Province China; 3 Department of Microbiology, School of Basic Medical Science, Central South University, Changsha, China; 4 China-Africa Research Center of Infectious Diseases, School of Basic Medical Sciences, Central South University, Changsha, Hunan Province China

**Keywords:** Gastrointestinal cancer, Non-coding RNAs, Epigenetics

## Abstract

Oxaliplatin is widely used in the frontline treatment of colorectal cancer (CRC), but an estimated 50% of patients will eventually stop responding to treatment due to acquired resistance. This study revealed that diminished MEIS1 expression was detected in CRC and harmed the survival of CRC patients. MEIS1 impaired CRC cell viabilities and tumor growth in mice and enhanced CRC cell sensitivity to oxaliplatin by preventing DNA damage repair. Mechanistically, oxaliplatin resistance following MEIS1 suppression was critically dependent on enhanced FEN1 expression. Subsequently, we confirmed that EZH2-DNMT3a was assisted by lncRNA ELFN1-AS1 in locating the promoter of MEIS1 to suppress MEIS1 transcription epigenetically. Based on the above, therapeutics targeting the role of MEIS1 in oxaliplatin resistance were developed and our results suggested that the combination of oxaliplatin with either ELFN1-AS1 ASO or EZH2 inhibitor GSK126 could largely suppress tumor growth and reverse oxaliplatin resistance. This study highlights the potential of therapeutics targeting ELFN1-AS1 and EZH2 in cell survival and oxaliplatin resistance, based on their controlling of MEIS1 expression, which deserve further verification as a prospective therapeutic strategy.

## Introduction

Colorectal cancer (CRC) is the third-most common cancer and the second leading cause of cancer-related death worldwide. By one estimate, there were 1.9 million cases of CRC and over 935,000 deaths in 2020.^1^ Most patients with early-stage CRC can be cured with surgery, but the prognosis of patients with advanced CRC at diagnosis is poor.^2^ At present, oxaliplatin with the combination of 5-fluorouracil and leucovorin (i.e., the FOLFOX regimen) is widely used as the first-line chemotherapeutic regimen for advanced CRC.^3^ However, the response is not satisfactory due to the dearth of effective predictive markers to identify patients who would gain benefits from such treatments.^4,5^ Although remarkable progress has been made in past decades, the molecular mechanisms of resistance to oxaliplatin in CRC remain poorly understood. Therefore, it is urgent to identify effective biomarkers and targets for the diagnosis and treatment of CRC.

Myeloid ecotype virus insertion site 1 (MEIS1), a member of the triple amino acid loop extension (TALE) family, plays a crucial role in cell development and proliferation. MEIS1 has been shown to act as an oncogene in leukemia but a tumor suppressor in other cancer types. The cancer-promoting role of MEIS1 involves promoting leukemogenesis and leukemic cell homing and enhancing cell-cell interactions and cell migration.^6^ Regarding its tumor-suppressive effects, MEIS1 inhibits the proliferation of non-small-cell lung cancer and prostate cancer cells;^7^ while the mechanism behind this tumor-suppressor role is largely unknown. In this study, we identified that MEIS1 impeded proliferation and oxaliplatin resistance of CRC by directly repressing flap structure-specific endonuclease 1 (FEN1), a key enzyme in the DNA damage repair pathway. In addition, our studies showed that the impaired expression of MEIS1 was highly correlated with poor prognosis of CRC patients.

Long noncoding RNAs (lncRNAs) are a class of noncoding transcripts over 200 nucleotides in length. Previous studies have shown that lncRNAs are aberrantly expressed in colorectal carcinoma and exert tumor-suppressive or oncogenic roles, depending on target genes.^8–10^ However, possible mechanisms underlying contribution of lncRNAs in tumor progression and oxaliplatin resistance of CRC need to be explored and further validated and analyzed. In this study, we clarified that ELFN1-AS1 could interact with enhancer of zeste homolog 2 (EZH2) and DNA methyltransferase 3 alpha (DNMT3a) which mediate trimethylation of Lys-27 in histone 3 (H3K27me3) and DNA methylation. We also proved that ELFN1-AS1 acted as a guide for the EZH2-DNMT3a complex to interact with the MEIS1 promoter region and subsequently suppress MEIS1 expression. Based on the above molecular mechanism, we developed a combination treatment comprising the ELFN1-AS1 ASO or EZH2 inhibitor GSK126 and oxaliplatin can significantly reverse the resistance of drug-resistant cells to oxaliplatin, impairing cell survival, and limiting tumor growth in mice. In summary, we determined prospective therapeutics targeting the role of MEIS1 in oxaliplatin resistance, and that ELFN1-AS1 or EZH2 can be potential targets for treating oxaliplatin-resistant CRC by promoting MEIS1 expression.

## Results

### Diminished MEIS1 expression is detected in CRC and harms the survival of CRC patients

**Fig. 1:**
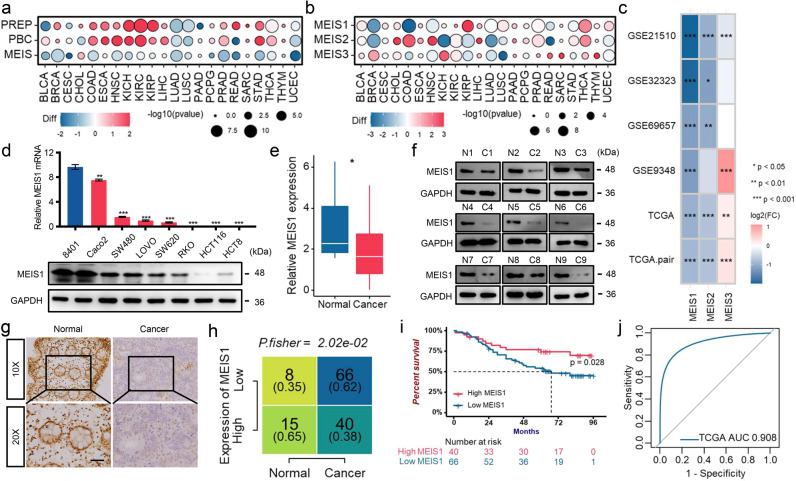
Diminished MEIS1 expression is detected in CRC and harms the survival of CRC patients. **a** The TALE subfamily score in paired samples grouped by cancer from TCGA. **b** Expression levels of MEIS family genes in paired samples grouped by cancer from TCGA. **c** Expression levels of MEIS family genes in the GEO and TCGA datasets. **d** qPCR (top) and western blot (bottom) of MEIS1 expression in the normal colon cell line 8401 and CRC cell lines. **e** qPCR analysis of MEIS1 expression in 12 normal colorectal and 44 CRC samples. **f** Western blot analysis of MEIS1 expression in 9 pairs of CRC samples and corresponding adjacent normal colorectal samples. **g**, **h** IHC of MEIS1 expression in CRC and adjacent nontumor samples. The data are presented as a representative image (**g**) (top, ×20 magnification; bottom, ×40 magnification) and summary graphs (**h**) (normal group, *n* = 23 and cancer group, *n* = 106). Scale bar, 100 mm. **i** Kaplan–Meier analysis showing overall survival (OS) curves of CRC patients stratified based on high versus low MEIS1 expression. **j** ROC curves displaying the sensitivity and specificity of MEIS1 for the diagnosis of CRC patients from the TCGA CRC dataset. Data are presented as the means ± SEM from three independent experiments. **p < 0.05, ****p < 0.0001

Although the TALE family members are involved in biochemical functions clearly intertwining with the cancer phenotypes, they are rarely reported to be the direct inducers of cancers.^11^ To explore basic features of TALE family members in cancers, we used single-sample gene set enrichment analysis (ssGSEA) to score three subfamilies of TALE-like genes (e.g., PREP, PBC, and MEIS) for describing their expression landscape in a variety of cancers compared to normal samples from The Cancer Genome Atlas (TCGA) (Fig. 1a and Supplementary Fig. S1a). What was noteworthy was that the relatively impaired expression of MEIS subfamily was consistent across multiple cancers, which conflicted with oncogenic effects of MEIS members reported in previous studies, with substantial evidence confirming its carcinogenic activity in hematologic tumors.^6^ Therefore, it is of interest to us whether the MEIS subfamily functions as a tumor-suppressor gene in solid tumors. Next, for determining which member contributed to the generally impaired expression of MEIS subfamily in cancers, we investigated the expression of MEIS1, MEIS2, and MEIS3 in multiple TCGA cancers (Fig. 1b and Supplementary Fig. S1b). With the aim of investigating tumor-suppress roles of MEIS members, we focused on MEIS1 with significantly diminished expression in TCGA Colon Adenocarcinoma (TCGA-COAD) dataset, which was further validated with external Gene Expression Omnibus (GEO) datasets including GSE21510,^12^ GSE32323,^13^ GSE9346,^14^ and GSE69657^15,16^ (a gene expression profiles of CRC patients treated with oxaliplatin chemotherapy) (Fig. 1c).

In accordance with TCGA and GEO datasets, the mRNA and protein levels of MEIS1 were significantly downregulated in CRC cell lines and tissues when compared to normal colon cell line 8401 cells and adjacent normal mucosal tissues, respectively (Fig. 1d–f). Immunohistochemistry (IHC) of CRC tissues also revealed impaired expression of MEIS1 (Fig. 1g, h). Besides, prognosis of CRC patients with relatively low expression of MEIS1 proved to be worse than those with high (Fig. 1i). Moreover, the area under the receiver operating characteristic (ROC) curve was performed to evaluate the diagnostic value of MEIS1 in TCGA dataset, which was 0.908 with a 95% confidence interval of 0.874–0.942 (*p* < 0.001), as shown in Fig. 1j. In summary, we identified uniquely downregulation of the MEIS subfamily in pan-cancer, especially MEIS1 in TCGA-COAD dataset, which was validated in external GEO datasets and CRC cell lines as well as CRC samples, and discovered that impaired expression of MEIS1 harmed the survival of CRC patients.

### MEIS1 impairs the viability and DNA replication of CRC cells and tumor growth in mice

**Fig. 2:**
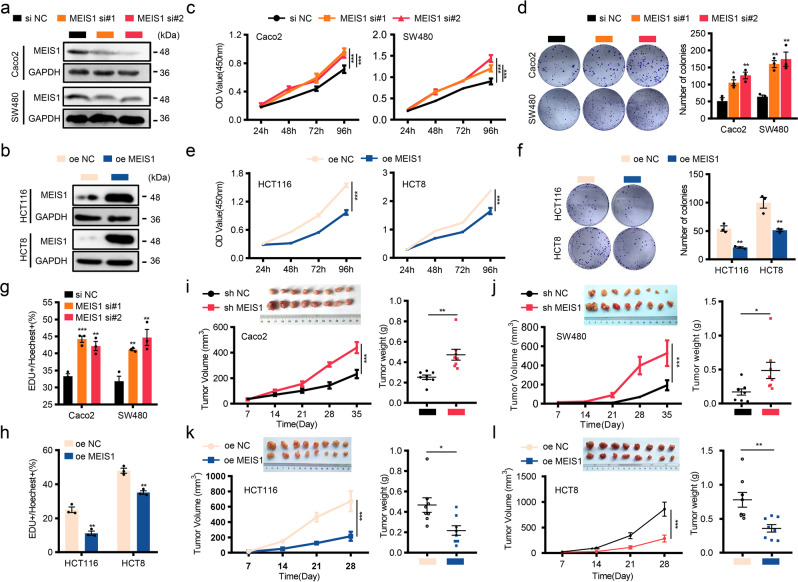
MEIS1 impairs the viability and DNA replication of CRC cells and tumor growth in mice. **a** Knockdown of MEIS1 in Caco2 and SW480 cells was confirmed by western blot. **b** Overexpression of MEIS1 in HCT116 and HCT8 cells was confirmed by western blot. **c**–**h** The proliferative ability of cells with MEIS1 knockdown or overexpression was determined by the CCK-8 assay (**c**, **d**), colony formation assay (**e**, **f**), and EdU assay (**g**, **h**). Scale bar: 50 μm. **i**–**l** Transplanted xenografts derived from cells with sh-MEIS1 and sh-NC (**i**, **j**) or oe-MEIS1 and oe-NC (**k**, **l**) were established in BALB/c nude mice (*n* = 8). Tumor volume and weight were measured (top-left, removed xenografts; bottom-left, tumor size; top-right, weight). The data are presented as the mean ± SEM from three independent experiments. *p < 0.05, **p < 0.01, ***p < 0.001

To investigate whether MEIS1 manipulates biological behaviors of CRC cells, we utilized two independent small interfering RNAs (siRNAs) to impair MEIS1 expression in CRC cell lines (Caco2 and SW480) with higher basal expression of MEIS1 (Fig. 2a), and elevated MEIS1 expression in cell lines (HCT116 and HCT8) with lower basal expression of MEIS1 (Fig. 2b). We could conclude from the results of CCK-8 and plate cloning assays that MEIS1-knockdown distinctly promoted viability and survival of Caco2 and SW480 cells (Fig. 2c, d), whereas overexpression of MEIS1 significantly suppressed these effects (Fig. 2e, f). The EdU assay confirmed the effect of MEIS1 on inhibition of DNA replication in CRC cell lines (Fig. 2g, h and Supplementary Fig. S2a, b). Similar results were also obtained in stable cell lines (Supplementary Fig. S2c–j).

Next, we utilized a nude mouse xenograft model to investigate the role of MEIS1 in CRC. Cell lines with stably knocked down or overexpressed MEIS1 were established and subcutaneously injected into nude mice in two independent experimental groups with their own controls. The tumor growth was subsequently measured and quantified. Consistent with previous in vitro studies, MEIS1 limited xenograft tumor growth, reflected by larger tumor mass and heavier tumor weight (Fig. 2i–l). IHC revealed that the ratio of Ki67-positive cells was negatively correlated with intracellular MEIS1 protein level, which supported that MEIS1 indeed impeded tumor growth (Supplementary Fig. S2k–l).

### MEIS1 enhances oxaliplatin sensitivity to regress CRC cells by preventing DNA damage repair

**Fig. 3:**
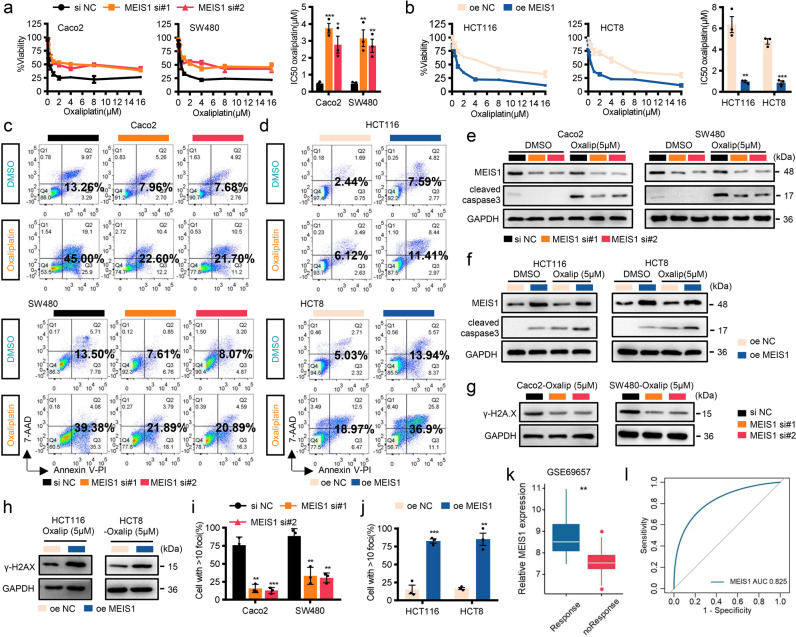
MEIS1 enhances oxaliplatin sensitivity to regress CRC cells by preventing DNA repair to trigger apoptosis. **a**, **b** si-MEIS1, oe-MEIS1, and corresponding control cells were treated with increasing concentrations of oxaliplatin for 48 h. Cell viability was determined by the CCK-8 assay (left), and the IC50 values were calculated based on nonlinear regression analysis (right). **c**–**f** Flow cytometry assays (**c**, **d**) and western blotting (**e**, **f**) were used to examine the effect of MEIS1 on the apoptosis of cells treated with oxaliplatin (5 μM) for 48 h. **g**, **h** Western blot was used to examine the γ-H2A.X levels in CRC cells treated with oxaliplatin (5 μM) for 24 h. **i**, **j** IF was used to examine γ-H2A.X foci in CRC cells treated with oxaliplatin (5 μM) for 24 h. **k** MEIS1 mRNA levels in samples from the GSE69657 dataset. Response, response to oxaliplatin; noResponse, no response to oxaliplatin. **l** ROC curves for predicting oxaliplatin resistance in the GSE69657 dataset. Data are presented as the mean ± SEM from three independent experiments. *p < 0.05, **p < 0.01, ***p < 0.001

Oxaliplatin, a platinum-based antineoplastic agent used in cancer chemotherapy, is able to promote apoptosis of tumor cells by inhibiting DNA synthesis, inducing DNA double-strand cross-linking, and blocking DNA replication and transcription processes.^17^ Previous bioinformatics analysis revealed that MEIS1 was significantly downregulated in CRC samples with oxaliplatin resistance (Fig. 1c), so we suspected that MEIS1 might be involved in the reactivity of CRC cells to oxaliplatin. Therefore, we assessed oxaliplatin sensitivity in CRC cell lines and discovered that CRC cells with lower levels of endogenous MEIS1 (HCT116 and HCT8 cell lines) were more resistant to oxaliplatin, with higher half maximal inhibitory concentration (IC50) values (Supplementary Fig. S3a). To further confirm that MESI1 contributed oxaliplatin sensitivity of CRC cells, we performed CCK-8, flow cytometry, and caspase-3 assays. Interestingly, after MEIS1 was knocked down, Caco2 and SW480 cells turned to be relatively resistant to oxaliplatin, exhibiting higher IC50 values and decrease apoptotic rate and cleaved caspase-3, while the opposite outcomes were observed in cells overexpressing MEIS1 (Fig. 3a–f). These results indicated that MEIS1 promoted the sensitivity of CRC cells to oxaliplatin.

To explore the impact of MEIS1 on oxaliplatin-induced DNA damage, we detected phospho-H2A.X (γ-H2A.X) levels and foci levels of oxaliplatin-treated CRC cells. Western blot assays indicated that abundant MEIS1 predisposed CRC cells to suffer DNA damage caused by oxaliplatin (Fig. 3g, h), according with the degree of γ-H2A.X foci detected by immunofluorescence (Fig. 3I, j and Supplementary Fig. S3b, c). It was worth noting that MEIS1 expression in patients who do not respond to oxaliplatin chemotherapy was significantly lower than that in responsive patients (Fig. 3k), which verified that MEIS1 contributed to accumulate DNA damage and thus enhanced oxaliplatin sensitivity. Furthermore, ROC curve analysis indicated that low MEIS1 expression might be a predictive marker to identify patients who are more likely to exhibit oxaliplatin resistance (Fig. 3l).

### Oxaliplatin resistance following MEIS1 suppression is critically dependent on enhanced FEN1 expression

**Fig. 4:**
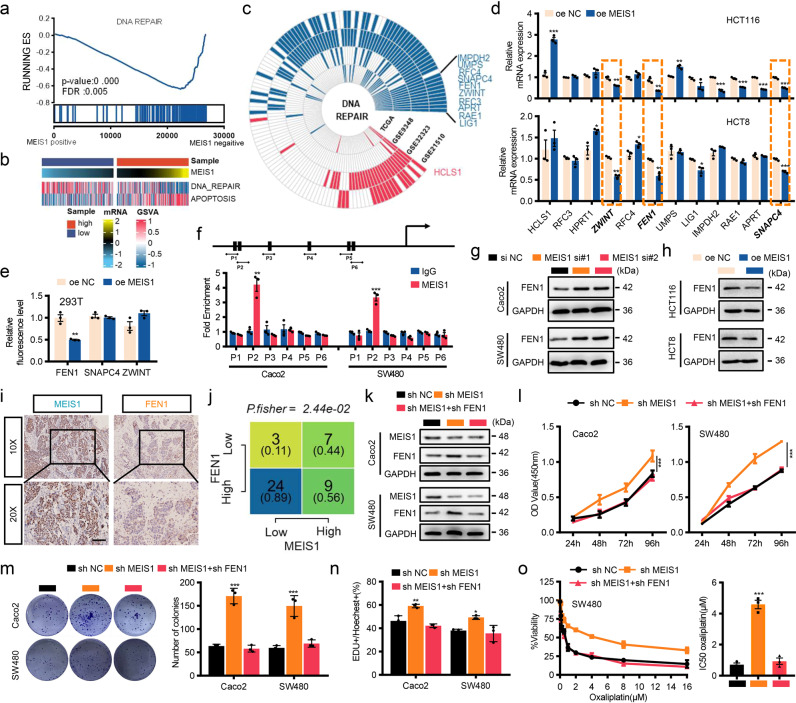
Oxaliplatin resistance following MEIS1 suppression is critically dependent on enhanced FEN1 expression. **a** GSEA plots showing that MEIS1 mRNA expression negatively correlated with DNA damage repair signatures in the TCGA datasets. **b** GSVA enrichment analysis showing the DNA repair and apoptosis pathways in the high- or low-MEIS1 groups. **c** Significant correlations were observed between MEIS1 expression and DNA damage repair pathway genes in the GSE9348, TCGA, GSE21510, and GSE32323 datasets. These genes were detected with | R | > 0.3 as the cutoff criterion in the four-cohort profile. Red represents positive correlations and blue represents negative correlations, Genes that were exclusively positive or negative correlations in all four datasets were screened as candidate genes. **d** qPCR results of the indicated RNA levels in cells transfected with oe-NC or oe MEIS1. **e** Luciferase reporter assay of the effect of FEN1, SNAPC4, and ZWINT on the transcriptional activity of the MEIS1 promoter. **f** Schematic showing putative MEIS1-binding sites on the promoter region of FEN1 and design of the indicated primers (top). ChIP-qPCR analysis of the enrichment of MEIS1 on the FEN1 promoter relative to that in control IgG-treated Caco2 and SW480 cells (bottom). **g**, **h** Representative western blot image of the FEN1 protein levels in cells with MEIS1 knockdown (**g**) or overexpression (**h**). **i**, **j** IHC of MEIS1 and FEN1 expression in CRC. Data are presented as a representative image (**i**) (top, ×20 magnification; bottom, ×40 magnification) and summary graphs(j). Scale bar, 100 µm. **k** Western blot analyses of MEIS1 and FEN1 expression in Caco2 and SW480 cells. **l**–**n** The proliferation of Caco-2 and SW480 cells was evaluated by the CCK-8 (**l**), cell colony formation (**m**), and EdU assays (**n**). **o** The cell viability and the IC50 values of SW480 cells was evaluated by the CCK-8 following increasing concentrations of oxaliplatin treatment for 48 h. The data are presented as the mean ± SEM from three independent experiments. *p < 0.05, **p < 0.01, ***p < 0.001

To further explore the mechanism underlying cell survival and high IC50 of oxaliplatin following MEIS1 suppression and identify target genes of MEIS1, we performed GSEA through ranking genes by Pearson correlation with the expression of MEIS1 in TCGA dataset, and discovered that the DNA REPAIR gene sets was significantly enriched (Fig. 4a). W Besides, we quantified DNA REPAIR and APOPTOSIS by standardized ssGSEA score. In general, those samples with high MSIE1 mRNA levels had relatively weak DNA damage repair process and severe apoptosis (Fig. 4b). To determine which target genes are regulated by MEIS1 in the DNA damage repair process, we screened DNA damage repair pathway genes co-expressing with MEIS1 in the TCGA and GEO cohorts (GSE21510, GSE32323, GSE9348) (*p* < 0.05 and | R | ≥ 0.3). HCLS1, positively associated MEIS1, and genes negatively associated MEIS1 including RFC3, HPRT1, ZWINT, RFC4, FEN1, UMPS, LIG1, IMPDH2, RAE1, APRT, and SNAPC4 were selected for further verification (Fig. 4c). Among them, FEN1, ZWINT, and SNAPC4 were confirmed by quantitative real-time PCR (qPCR) to be significantly downregulated by MEIS1 at mRNA levels (Fig. 4d).

Considering the transcriptional regulation role of MEIS1 and our aim of revealing the direct target of MEIS1, we analyzed promoter regions of FEN1, ZWINT, and SNAPC4 in JASPAR database and discovered that all of them contained potential MEIS1 binding sites. Dual-Luciferase reporter system verified that MEIS1 could significantly reduce the reporter activity driven by the FEN1 promoter but not that of ZWINT or SNAPC4 (Fig. 4e). In addition, chromatin immunoprecipitation (ChIP) assays confirmed that MEIS1 could directly bind to the promoter regions of FEN1 gene at MEIS1-binding-site-2 (Fig. 4f). Western blot verified that MEIS1 was able to downregulate FEN1 expression at protein level in CRC cells (Fig. 4g, h), and under treatment with oxaliplatin (Supplementary Fig. S4a, b). Besides, at both mRNA and protein levels, expressions of FEN1 were enhanced in CRC tissues and negatively correlated with MEIS1 in our cohort, which were confirmed by qPCR (Supplementary Fig. S4c, d) and IHC (Fig. 4I, j). Moreover, patients who tended not to respond to oxaliplatin were with relatively high FEN1 expression CRC and significantly negative correlation was existed between MEIS1 and FEN1 (Supplementary Fig. S4e–f). Particularly, FEN1 activation was required for strengthened cell viability and survival of CRC cells and relatively high IC50 of oxaliplatin following MEIS1 suppression (Fig. 4k–o). In summary, MEIS1 interfered with cell survival and rendered cell sensitive to oxaliplatin by directly targeting and negatively regulating FEN1.

### Epigenetic silencing maintained by the EZH2/DNMT3a complex contributes to suppressed transcription of MEIS1 in CRC

**Fig. 5:**
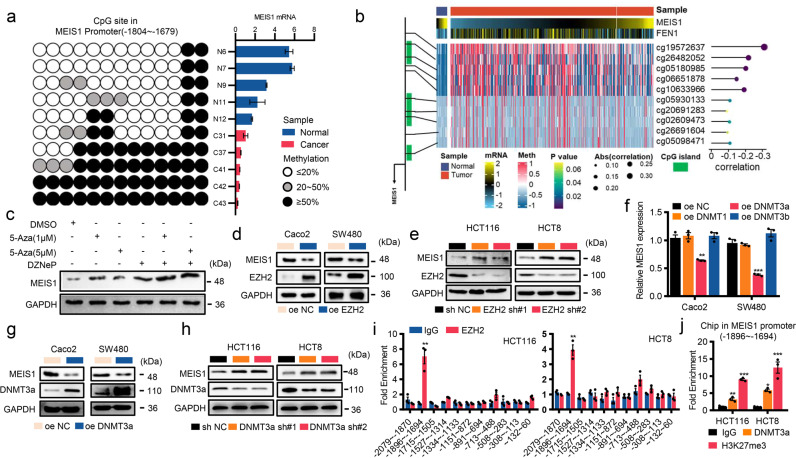
Epigenetic silencing maintained by the EZH2/DNMT3a complex contributes to suppressed transcription of MEIS1 in CRC. **a** BSP analysis of CpG methylation sites on the promoter region of MEIS1 in CRC tumor specimens. The color inside the circle represents the average CpG island methylation status obtained from five independent clones (left). The expression of MEIS1 was analyzed by qPCR (right). **b** Schematic showing the CpG islands of MEIS1 (left), CpG islands (green) and probes (black line). Heatmap with beta values of DNA methylation obtained from ten MEIS1 probes in normal colorectal tissues and CRC tumors from the TCGA 450K array (middle). Correlation analysis of MEIS1 probes and MEIS1 expression levels by the Pearson correlation coefficient (right). **c** Western blot analysis of MEIS1 expression in HCT116 cells treated with different combinations of epigenetic drugs. **d**, **e** Western blot analyses of EZH2 and MEIS1 expression in CRC cell lines transfected with EZH2 shRNA or oe-EZH2, and their corresponding controls. **f** qPCR analysis of MEIS1 mRNA levels in Caco2 and SW480 cells transfected with oe-NC, oe-DNMT1, oe-DNMT3a, or oe-DNMT3b. **g**, **h** Western blot of DNMT3a and MEIS1 expression in CRC cell lines transfected with DNMT3a shRNA, oe-DNMT3a, and their corresponding controls. **i** ChIP-qPCR analysis of the enrichment of EZH2 on the MEIS1 promoter relative to that of control IgG in HCT116 and HCT8 cells. **j** ChIP-qPCR analysis of the DNMT3a genomic occupancy and H3K27 methylation status at the MEIS1 promoter. Data are presented as the mean ± SEM from three independent experiments. *p < 0.05, **p < 0.01, ***p < 0.001

Analysis of data with cBioPortal (http://www.cbioportal.org/) revealed that the incidence of deletion mutation of MEIS1 was not significant in CRC (Supplementary Fig. S5a), suggesting that there are some other mechanisms responsible for the impaired expression of MEIS1 in CRC. Several homeobox-containing genes were aberrantly expressed due to alterations in the methylation profiles of promoters.^18^ Therefore, we suspected that epigenetic modification might contribute to the impaired expression of MEIS1. Next, we identified three CpG islands in the promoter region of MEIS1 and designed bisulfite PCR primer using MethPrimer software (Supplementary Fig. S5b). Bisulfite sequencing revealed that low MEIS1 mRNA levels in CRC samples were accompanied by hypermethylated promoter regions of MEIS1 (Fig. 5a), which supported by correlation analyses based on TCGA dataset (Fig. 5b and Supplementary Fig. S5c, d). Moreover, it has been shown that the BRAF^V600E^ mutation is related to the methylation of MEIS1 in CRC.^19^ We analyzed the association between BRAF mutation and MEIS1 in TCGA dataset and discovered that the promoter regions of MEIS1 were all hypermethylated compared to normal, regardless of the presence or absence of BRAF mutations, and the methylation level of MEIS1 promoter regions is the highest in BRAF^p.V600E^ group (Supplementary Fig. S5e).

Previous studies have shown that EZH2 interacts with DNA methyltransferases (DNMTs) to methylate DNA and remodel chromatin.^20^ After treating HCT116 cells with 3-deazaneplanocin A (DZNeP), a histone methyltransferase inhibitor, and 2′-deoxy-5-azacytidine (5-Aza), a DNMT inhibitor, we detected the expression level of MEIS1. 5-Aza or DZNeP alone could upregulate MEIS1 expression, but 5-Aza in combination with DZNeP greatly enhanced the upregulation (Fig. 5c). Furthermore, the significant negative regulation of EZH2 on MEIS1 had also been elucidated in cell (Fig. 5d, e). As a nucleoside analog of cytidine, 5-Aza is able to specifically inhibit DNA methylation by covalently bonding to DNMTs and blocking their catalytic activity. Through manipulating the expression of DNMTs in CRC cells, we discovered that only DNMT3a could notably regulate the expression of MEIS1 (Fig. 5f–h).

Since EZH2 functions by binding to the promoter of its target gene, ChIP was performed to confirm that EZH2 was significantly localized to a specific region (−1896 ~ −1694 nt) of the MEIS1 promoter (Fig. 5i), and DNMT3a and H3K27me3 were likewise enriched in this region (Fig. 5j). In general, our results strongly suggested that epigenetic silencing of MEIS1 in CRC involved EZH2 and DNMT3a.

### EZH2/DNMT3a is assisted by lncRNA ELFN1-AS1 in locating the promoter of MEIS1 to suppress MEIS1 expression

**Fig. 6:**
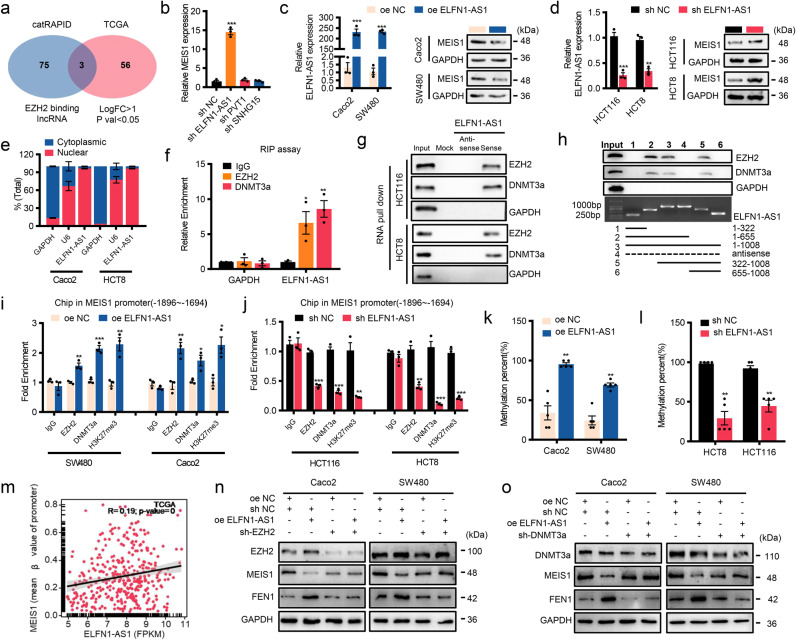
EZH2/DNMT3a is assisted by lncRNA ELFN1-AS1 in locating the promoter of MEIS1 to suppress MEIS1 expression. **a** Venn diagram between EZH2-binding lncRNAs identified by catRAPID and upregulated lncRNAs in the TCGA CRC dataset (*p* < 0.05 and | logFC | ≥ 1). **b** qPCR analysis of MEIS1 expression levels in CRC cells transfected with shRNAs targeting lncRNAs. **c**, **d** qPCR and western blot analysis of the ELFN1-AS1 mRNA and MEIS1 protein levels, respectively, were determined in cells with overexpression (**c**) or knockdown of ELFN1-AS1 (**d**). **e** After separation of the nuclear and cytosolic fractions, the RNA expression levels were measured by qPCR. GAPDH was used as a cytoplasmic control gene, and U6 was used as the nuclear control gene. Data are presented as percentages of GAPDH, U6, and ELFN1-AS1 levels, and total levels for each were taken as 100%. **f** RIP experiments for IgG, EZH2, and DNMT3a were performed, followed by qPCR to examine the enrichment of ELFN1-AS1. **g**, **h** The interaction of full-length (**g**) and truncated (**h**) ELFN1-AS1 with EZH2 and DNMT3a was confirmed by RNA pull-down and immunoblotting. **i**, **j** ChIP-qPCR analysis of the enrichment of EZH2, DNMT3a and H3K27 methylation status at the MEIS1 promoter in CRC cells with overexpression (**i**) or knockdown (**j**) of ELFN1-AS1. **k**, **l** BSP analysis of the DNA methylation status of the MEIS1 locus in CRC cells with overexpression (**k**) or knockdown (**l**) of ELFN1-AS1. **m** Correlation of ELFN1-AS1 expression with the MEIS1 DNA methylation status in CRC and normal tissues from the TCGA 450K array by the Pearson correlation coefficient. **n** Western blot of EZH2, MEIS1, and FEN1 expression in Caco2 and SW480 cells transfected with EZH2 shRNA, oe-ELFN1-AS1, and their corresponding controls. **o** Western blot of DNMT3a, MEIS1 and FEN1 expression in Caco2 and SW480 cells transfected with DNMT3a shRNA, oe-ELFN1-AS1, and their corresponding controls. Data are presented as the mean ± SEM from three independent experiments. *p < 0.05, **p < 0.01, ***p < 0.001

Multiple studies have indicated that various lncRNAs can lead EZH2 to locate on target genes specifically, regulating their transcription transcription.^21,22^ Therefore, we took into account 78 lncRNAs binding to EZH2 predicted by catRAPID and 59 upregulated lncRNAs in CRC samples from TCGA dataset and intersected them to obtain upregulated lncRNAs binding to EZH2, including ELFN1-AS1, PVT1, and SNHG15 (Fig. 6a). Among them, only knockdown of ELFN1-AS1 significantly elevated mRNA level of MEIS1 (Fig. 6b), whose protein levels were also regulated with additional or deprivation of ELFN1-AS1 (Fig. 6c, d). Meanwhile, correlation analysis between ELFN1-AS1 and MEIS1 as well as FEN1 performed in our cohort and public datasets further supported above results (Supplementary Fig. S6a).

ELFN1-AS1 is transcribed from the intron of the ELFN1 gene located on human chromosome 7p22.3 (Supplementary Fig. S6b). Analysis based on the coding potential calculator (CPC) and Coding Potential Assessment Tool (CPAT) software indicates that ELFN1-AS1 is a noncoding RNA similar to the better known lncRNA CCAT1^23^ (Supplementary Fig. S6c). Nucleocytoplasmic separation assay indicated enrichment of ELFN1-AS1 in the nuclei of CRC cells (Fig. 6e). Given that nuclear-retained lncRNAs involve the transcription and epigenetic regulation of genes, and potential interactions between ELFN1-AS1 and EZH2 as well as DNMT3a based on CatRAPID algorithm and RNA-protein interaction prediction (RPISeq) (Supplementary Fig. S6d), we suspected that, in the process of regulating MEIS1 transcription, ELFN1-AS1 might interact with EZH2 and DNMT3a, which was confirmed by RNA immunoprecipitation (RIP) (Fig. 6f) and RNA pull-down assays (Fig. 6g). In addition, we constructed a series of truncated ELFN1-AS1 mutants and identified that the 322–655 nt fragment of ELFN1-AS1 was necessary to bind EZH2 and DNMT3a (Fig. 6h). Taken together, these results indicated that ELFN1-AS1 could directly bind to EZH2 and DNMT3a.

Previous results confirmed that EZH2, DNMT3a, and ELFN1-AS1 were involved in regulating MEIS1 transcription and that ELFN1-AS1 could interact with EZH2 as well as DNMT3a. We suspected that ELFN1-AS1 exerted its function by recruiting EZH2 and DNMT3a to the promoter region of MEIS1. ChIP-qPCR assay confirmed that ELFN1-AS1 assisted enrichment of EZH2 and DNMT3a in the potential promoter region of MEIS1(−1896 ~ −1694), elevating H3K27me3 and DNA methylation levels (Fig. 6i–l). Besides, the significantly positive correlation between mRNA level of ELFN1-AS1 and the methylation level of the promoter of MEIS1 in TCGA dataset supported above discoveries (Fig. 6m). Further, the effect of ELFN1-AS1 on the expression and the function of MEIS1 was dependent on the presence of EZH2/DNMT3a (Fig. 6n–o). Meanwhile, we wondered that whether ELFN1-AS1 promoted the expression of EZH2 and DNMT3a or was elevated by them, which proved to be false subsequently (Supplementary Fig. S6e–g). Taken together, we identified a lncRNA, interacting with EZH2 as well as DNMT3a and enriching them in the promoter region of MEIS1, contributed DNA methylation and H3K27me3 in the promoter region of MEIS1, thereby suppressing MEIS1 transcription and the expression of its downstream FEN1.

### LncRNA ELFN1-AS1 promotes tumor growth and oxaliplatin resistance by suppressing MEIS1 expression

**Fig. 7:**
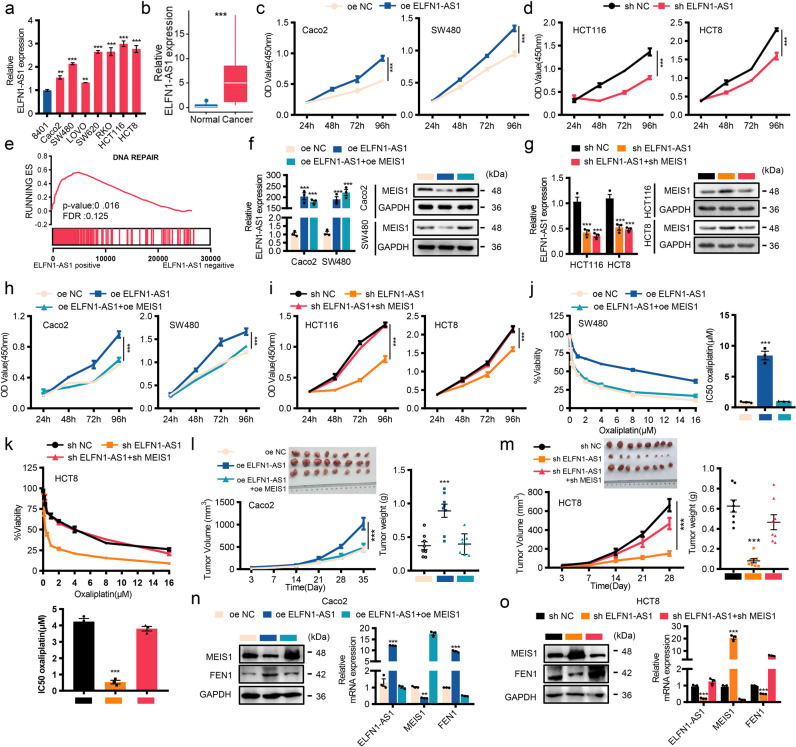
LncRNA ELFN1-AS1 promotes tumor growth and oxaliplatin resistance by suppressing MEIS1 expression. **a** qPCR of ELFN1-AS1 mRNA levels in CRC cells and the normal cell line 8401. **b** qPCR analysis of ELFN1-AS1 expression in 12 normal colorectal samples and 44 CRC samples. **c**, **d** CRC cell proliferation was evaluated by the CCK-8 assay. **e** GSEA plots showing that ELFN1-AS1 mRNA expression was positively correlated with DNA damage repair signatures in the TCGA CRC datasets. **f**, **g** The ELFN1-AS1 mRNA and MEIS1 protein levels were determined by qPCR and western blot, respectively. **h**, **i** CRC cell proliferation was determined by the CCK-8 assay. **j**, **k** The cell viability and the IC50 values of SW480 and HCT8 cells was evaluated by the CCK-8 following increasing concentrations of oxaliplatin treatment for 48 h. **l**, **m** Xenograft tumors derived from Caco2 cells with oe-ELFN1-AS1, oe-ELFN1-AS1 + oe MEIS1 and oe-NC (**l**) or HCT8 cells with sh-ELFN1-AS1, sh-ELFN1-AS1 + sh-MEIS1 and sh-NC (**m**) in BALB/c nude mice (*n* = 8). Tumor volume and weight were measured (top-left, removed xenografts; bottom-left, tumor size; top-right, weight). **n**, **o** The expression of MEIS1 and FEN1 in xenograft tumors was detected by western blot and qPCR. Data are presented as the mean ± SEM from three independent experiments. ***p < 0.001

Relatively high mRNA levels of ELFN1-AS1 were detected in CRC tissues and cell lines (Fig. 7a, b) and verified by public datasets (Supplementary Fig. S7a), and ROC curve analysis indicated that ELFN1-AS1 combined with MEIS1 was superior to MEIS1 alone for diagnosing CRC (Supplementary Fig. S7b, c). To adequately evaluate the biological function of ELFN1-AS1 in CRC, we manipulated the levels of ELFN1-AS1 and discovered that overexpressing ELFN1-AS1 alone was able to enhance cell viability, maintain cell survival, and impair sensitivity of CRC cells to oxaliplatin (Fig. 7c, d, Supplementary Fig. S7d–i). Interestingly, GSEA indicated that DNA REPAIR related genes were also significantly enriched and positively correlated with ELFN1-AS1 in TCGA dataset (Fig. 7e).

Considering the negative correlation between MEIS1 and DNA damage repair pathway and its downstream FEN1, we hypothesized that ELFN1-AS1 exerted its biological functions by regulating MEIS1. Therefore, we replenished or weakened the changes in MEIS1 caused by manipulations of ELFN1-AS1 (Fig. 7f, g), and investigated biological functions of ELFN1-AS1 (Fig. 7h, i, Supplementary Fig. S7j–m), and its effect on sensitivity to oxaliplatin (Fig. 7j, k). Besides, the negative correlation existed between ELFN1-AS1 and MEIS1 in patients who did not response to oxaliplatin (Supplementary Fig. S7n). In vivo assay indicated that the effect of ELFN1-AS1 on tumor growth depends on its negative regulation of MEIS1 (Fig. 7l, m), verified and supported by detecting these mRNA and protein levels derive from xenograft tumor samples (Fig. 7n, o). Above data suggested a presumption of MEIS1 regulated by ELFN1-AS1 possibly mediating oxaliplatin resistance.

### The EZH2 inhibitor GSK126 sensitizes CRC cells to oxaliplatin

Given that the impaired expression of MEIS1 contributed to elevated IC50 of oxaliplatin and the essential role of ELFN1-AS1/EZH2-DNMT3a in controlling MEIS1 transcription, we attempted to transform this mechanism to pave a new way for clinical treatment of oxaliplatin resistant CRC patients.

**Fig. 8:**
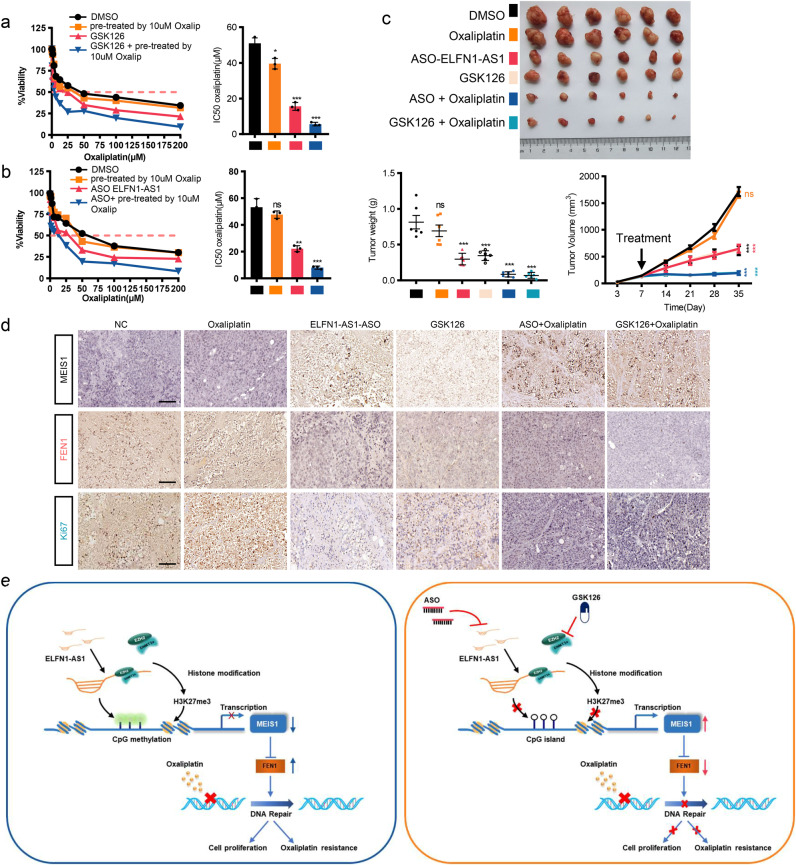
The EZH2 inhibitor GSK126 or lncRNA ELFN1-AS1 ASO sensitizes HCT116/L-OHP to oxaliplatin. **a** The IC50 values of oxaliplatin in HCT116/L-OHP cells treated with oxaliplatin (10 μM), GSK126, GSK126 + oxaliplatin (10 μM) and DMSO. **b** The IC50 values of oxaliplatin in HCT116/L-OHP cells treated with oxaliplatin (10 μM), ASO-ELFN1-AS1, ASO + oxaliplatin (10 μM) and DMSO. **c** HCT116/L-OHP cells were inoculated into four-week-old male BALB/C nude mice. When the tumors became palpable, they were injected with 0.1% DMSO, oxaliplatin, ASO-ELFN1-AS1, GSK126, ASO-ELFN1-AS1 + oxaliplatin or GSK126 + oxaliplatin. The tumor volume and weight were measured. **d** IHC of MEIS1, FEN1 and Ki67 in the tumors. **e** Schematic representation of a model for the major molecular mechanisms (left) and drug intervention hypothesis (right) for the ELFN1-AS1-EZH2-DNMT3a/MEIS1/FEN1 axis in CRC. Data are presented as the mean ± SEM from three independent experiments. *p < 0.05, **p < 0.01, ***p < 0.001, ****p < 0.0001, ns, no significance

First, we authenticated the oxaliplatin resistance characteristics of oxaliplatin-resistant HCT116/L-OHP cells relative to parental HCT116 cell lines via comparing IC50 of oxaliplatin and cell viability, survival and apoptosis rates between HCT116/L-OHP and HCT116 with or without oxaliplatin (Supplementary Fig. S8a–d). We also observed impaired expression of MEIS1 and enhanced EZH2, DNMT3a, and FEN1 in HCT116/L-OHP cells (Supplementary Fig. S8e, f), and MEIS1 supplementation could reverse oxaliplatin resistance of HCT116/L-OHP cells (Supplementary Fig. S8g–j), suggesting that MEIS1 could benefit to reverse oxaliplatin resistance. Therefore, we looked for small molecules developed already potentially upregulating MEIS1 and then focused on inhibitors of EZH2.^24^ Among them, GSK126 with a highly selective and inhibitory effect on EZN2 was investigated further in this study.^25^ GSK126 did increased the protein level of MEIS1 in HCT116/L-OHP cells in a dose-dependent manner (Supplementary Fig. S9a). Meanwhile, GSK126 exerted strongly inhibitory effect on oxaliplatin-resistant cells depending on the upregulation of MEIS1 (Supplementary Fig. S9b–e), and affected on oxaliplatin sensitivity similarly as MESI1 overexpression did in vitro (Supplementary Fig. S9f). Next, we wondered whether GSK126 could reverse oxaliplatin resistance. When oxaliplatin alone was ineffective or slightly effective, the application of oxaliplatin combined with GSK126 sensitized HCT116/L-OHP cells with weakened cell viability and elevated apoptosis rate (Fig. 8a and Supplementary Fig. S9g–i, k). And the combination of GSK126 with oxaliplatin was synergistic (Supplementary Fig. S9j).

### LncRNA ELFN1-AS1 ASO reverses the resistance of HCT116/L-OHP to oxaliplatin

In recent years, ASO drugs have obtained considerable attentions due to their ability to target RNAs more precisely compared to small molecule compounds, which has been verified in vivo and in vitro.^26,27^ Here, we aimed to explore the value of ASOs in the treatment of oxaliplatin resistance CRC patients.

First, we designed two ASOs specific for ELFN1-AS1 as well as a negative control, and only ASO-2 exerting suppression on the mRNA level of ELFN1-AS1 was selected for subsequent analysis (Supplementary Fig. S10a). ASO-2 was confirmed to enhance MEIS1 expression without disturbing EZH2 (Supplementary Fig. S10b), and did exert strongly inhibitory effect on HCT116/L-OHP cells depending on MEIS1 (Supplementary Fig. S10c–f). ELFN1-AS1 ASO affected on oxaliplatin sensitivity similarly as MESI1 overexpression did in vitro (Supplementary Fig. S10g), and ELFN1-AS1 ASO combined with oxaliplatin could improve the reactivity of resistant cells to oxaliplatin (Fig. 8b and Supplementary Fig. S10h–j, l). And the combination of ASO with oxaliplatin was synergistic (Supplementary Fig. S10k). To investigate the role of the ELFN1-AS1/EZH2-MEIS1 axis in regulating oxaliplatin resistance in CRC in vivo, HCT116/L-OHP cells were inoculated into four-week-old male BALB/C nude mice. Mice were treated when tumors became palpable (Supplementary Fig. S10m). Consistent with the in vitro results, oxaliplatin alone had limited effect on tumor suppression. Notably, treatment with either ASO-ELFN1-AS1 or GSK126 significantly decreased tumor volumes, and combination of either agent with oxaliplatin further prevented tumor growth, suggesting that ASO-ELFN1-AS1 and GSK126 inhibited tumor growth and restore sensitivity of oxaliplatin-resistant CRC tumors to oxaliplatin (Fig. 8c). IHC showed that MIES1 expression was increased after ELFN1-AS1-ASO or GSK126 treatment, while the expression of Ki67 and FEN1 was markedly lower in tumors treated with ELFN1-AS1-ASO or GSK126 and nearly deprived with addition of oxaliplatin (Fig. 8d).

Taken together, this study elaborates potential mechanisms underlying diminished MEIS1 in CRC and its contribution in oxaliplatin resistance. Oxaliplatin resistance following MEIS1 suppression is critically dependent on enhancive FEN1 expression. Impaired expression of MEIS1 is mediated by EZH2-DNMT3a assisted with lncRNA ELFN1-AS1 locating in the promoter of MEIS1 and suppressing MEIS1 transcription epigenetically. MEIS1 could enhance viability of CRC cells and tumor growth in mice and enhance oxaliplatin sensitivity to regress CRC cells by preventing DNA damage repair. Therefore, therapeutics targeting the role of MEIS1 in oxaliplatin resistance are developed and our results suggest that the combination of oxaliplatin with either ELFN1-AS1 ASO or EZH2 inhibitor GSK126 could largely suppress tumor growth and reverse oxaliplatin resistance (Fig. 8e).

## Discussion

The TALE proteins is a class of transcription factors characterized by homeodomain (HD), which is a triple-helix DNA-binding domain consisting of about 60 amino acid residues.^28^ Though the TALE family members are involved in biochemical functions clearly intertwining with cancer phenotypes, their function in cancers remains unclear. Herein, we used ssGSEA to describe the landscape of mRNA levels of three subfamilies composed of TALE-like genes in a pan-cancer array and discovered impaired expression level of the whole MEIS subfamily in various human cancers, especially the significantly downregulation of subfamily member MEIS1 in TCGA-COAD. Characteristics of MEIS1 in leukemia, prostate cancer, and axial embryo models have already been elucidated.^6,7,29^ However, there is debate when it comes to the role of MEIS1 in solid tumors. The controversy is reflected in the potential tumor-suppress role of MEIS1 in prostate cancer^30^ and clear cell renal cell carcinoma^31^ and that depletion of MEIS1 as well as MEIS2 enhanced tumor growth by upregulating c-MYC and CD142,^7^ but the oncogenic role of MEIS1 in glioma.^32,33^ Therefore, the functions of MEIS1 in different solid tumors need to be further elaborated. Previous research has revealed that the expression level of MEIS1^D27^, a truncated splicing variant of MEIS1, decreased in primary colorectal cancer samples, insinuating tumor suppressive effect of MEIS1,^34^ which is consistent with our results obtained in CRC samples from public datasets, especially in GSE69657 composed of oxaliplatin resistant CRC patients. Similarly, significantly impaired mRNA and protein levels of MEIS1 were verified in CRC cell lines and tissues and contributed to enhanced cell viability, tumor growth in mice, and oxaliplatin resistance.

The FOLFOX is widely used as the first-line chemotherapeutic regimen of primarily colorectal cancer. However, the response is not satisfactory due to the dearth of effective predictive markers of sensitivity to treatment.^4,5^ In this study, ROC curve analysis indicated that MEIS1 had the potential to identify patients inclined to be resistant to oxaliplatin. Further inquiry into the mechanisms of oxaliplatin resistance is crucial to optimizing current treatment strategies. In general, oxaliplatin induces the formation of inter- and intrastrand DNA-platinum adducts and then inhibits gene transcription by segregating transcription factors, thus leading to apoptosis.^35^ So tumors fight back by enhancing DNA damage repair, resulting in oxaliplatin resistance and severely limits efficacy of other chemotherapy drugs.^36^ We suggested that enhancing the expression of MEIS1 could prevent the activation of the DNA damage repair. FEN1, a structure-specific endonuclease with 5′-exonuclease and endonuclease activity,^37^ plays multiple roles in processing intermediates of Okazaki fragment maturation, telomere maintenance and stalled replication fork rescue.^38^ Generally enhanced expression of FEN1 in tumors may lead to chemoresistance.^39–41^ Our results indicated that FEN1 was highly expressed in CRC and the effect of MEIS1 on tumor growth and oxaliplatin reactivity depended on FEN1 suppression.

Based on the above, we turned to seek for targetable molecules that was sufficient to simulate effects of MEIS1 including tumor suppression and enhancing oxaliplatin sensitivity to regress CRC cells. Given that homeobox-containing genes were aberrantly expressed due to alterations in the methylation profiles of promoters,^18^ we next took the epigenetic regulation of MEIS1 as the focus for further exploration. Moreover, it has been shown that the BRAF^V600E^ mutation is related to the methylation of MEIS1 in CRC.^19^ In this study, almost all CRC samples exhibited hypermethylated promoter regions of MEIS1, regardless of BRAF was mutated or not, and the methylation level is the highest in BRAF^p.V600E^ group in TCGA dataset and our cohort.

EZH2 is a histone methyltransferase subunit of Polycomb Repressive complex 2, which mediates H3K27me3 and acts as a key oncogene for tumor proliferation, metastasis and drug resistance.^42,43^ Previous studies have shown that EZH2 interacts with DNMTs to methylate DNA and remodel chromatin.^20^ DNMT3a is a de novo DNA methyltransferase that can actively add new methyl groups to DNA sequences to control gene expression^44^ and plays a vital role in the carcinogenic process.^45^ In addition, EZH2 interacts with DNMTs, resulting in DNA methylation and chromatin remodeling.^20^ Either elevating the expression of EZH2 or DNMT3a significantly suppressed the transcription of MEIS1. Further, ChIP assay confirmed direct interactions between MEIS1 promoter region (-1804 ~ -1679 nt) and EZH2 as well as DNMT3a.

By the way, there is an interesting phenomenon here. MEIS1 expression was slightly increased by 5-Aza, but strongly increased expression in cells treated with 5-Aza and DZNeP (EZH2 inhibitor) together. Therefore, we believed that the increase in MEIS1 expression is affected by the synergistic effect of DZNeP and 5-Aza, and DNMT3a/EZH2 complex is more important for the transcriptional repression of MEIS1expression than each of them alone. This point was also supported by the result of Fig. 5i which showed that knocking down of DNMT3a alone could significantly enhance MEIS1 expression. Given that both DNMT3a and EZH2 were involved in the inhibition of MEIS1 expression, knocking down DNMT3a alone might result in transcriptional complex incomplete which could not repress MEIS1 expression efficiently. We highly speculated that 5-Aza can only inhibit the enzymatic activity of DNMTs, and cannot completely block the formation of transcriptional complex between DNMT3a and EZH2. Therefore, overexpression of DNMT3a may have a greater influence on MEIS1 expression than blocking catalytic activity of DNMT.

Among manifold biological functions of lncRNAs,^22^ it is one of the most essential that directing and recruiting histone-modifying enzymes or transcription factors to specific genomic sites and thus regulating gene transcription^46^ Multiple studies have indicated that various lncRNAs can lead EZH2 to locate on target genes specifically, regulating their transcription.^21,22^ Previous studies have shown that POU3F3 adjacent non-coding transcript 1, ncRNA (PANTR1) promoted the methylation of POU3F3 by interacting with EZH2, thereby promoting the occurrence of ESCC.^47^ LINC00673 inhibited KLF4 expression in gastric cancer cells by interacting with EZH2 and DNMT1.^48^ In this study, relatively high mRNA levels of ELFN1-AS1 were detected in CRC tissues and cell lines, and overexpressing ELFN1-AS1 alone was able to enhance cell viability and impair sensitivity of CRC cells to oxaliplatin. Mechanistically, we identified that a lncRNA ELFN1-AS1, interacting with EZH2 as well as DNMT3a and enriching them in the promoter region of MEIS1, contributed DNA methylation and H3K27me3 in the promoter region of MEIS1.

Identifying patients who might respond well to treatment and tolerate adverse events is the primary problem to be solved in personalized treatment strategies. Recently, combinations of conventional chemotherapeutic agents with compounds were reported and have proven to be effective in synergistically restricting tumor growth and reducing side effects caused by toxicity.^49,50^ Based on above principles, therapeutics targeting the ELFN1-AS1/EZH2-DNMT3a/MEIS1 axis are developed for oxaliplatin resistant CRC patients, and our results suggest that the combination of oxaliplatin with either ELFN1-AS1 ASO or EZH2 inhibitor GSK126 could largely suppress tumor growth and reverse oxaliplatin resistance in vivo and in vitro. This combination regimen deserves further validation in clinical and may exert positive effects on improving the prognosis of oxaliplatin resistant patients.

However, there are still limitations and questions in suspense needed to be further investigated. In the study of combination regimens in reversing oxaliplatin resistance, we only used one oxaliplatin-resistant cell line to evaluate therapeutic effect, and actually, we should use more to support our conclusions. Moreover, ELFN1-AS1/EZH2/DNMT3a affected tumor growth and oxaliplatin resistance by regulating MEIS1-FEN1. Whether MEIS1 involves DNA damage repair through mediating FEN1 needs further study.

In summary, this study elaborates potential mechanisms underlying diminished MEIS1 in CRC and its contribution in oxaliplatin resistance. Oxaliplatin resistance following MEIS1 suppression is critically dependent on enhancive FEN1 expression. Impaired expression of MEIS1 is mediated by EZH2-DNMT3a assisted with lncRNA ELFN1-AS1 locating in the promoter of MEIS1 and suppressing MEIS1 transcription epigenetically. MEIS1 could enhance viability of CRC cells and tumor growth in mice and enhance oxaliplatin sensitivity to regress CRC cells by preventing DNA damage repair. This study highlights the potential of therapeutics targeting ELFN1-AS1 or EZH2 in cell survival and oxaliplatin resistance, based on their controlling of MEIS1 expression, which deserve further verification as a prospective therapeutic strategy.

## Materials and methods

### Cell lines and cell culture

The human embryonic kidney cell line HEK-293T (ATCC CRL-3216) and CRC cell lines HCT116 (ATCC CCL-247), HT29 (ATCC HTB-38), SW480 (ATCC CCL-228), and SW620 (ATCC CCL-227) were purchased from American Type Culture Collection (ATCC; http://www.atcc.org/). HCT116/L-OHP cells with oxaliplatin resistance were purchased from MEIXUAN Biological Science & Technology (Shanghai, China). The CRC cell lines HCT8, Caco2, RKO and LOVO were kindly provided by Professor Wancai Yang (Key Laboratory of Precision Oncology of Shandong Higher Education, Institute of Precision Medicine, Jining Medical University). The normal colon epithelial cell line 8401 (also known as CCD 841 CoN, ATCC® CRL­ 1790™) was kindly provided by Professor Lunquan Sun (Xiangya Hospital, Central South University, Changsha, China). HCT116 cells were maintained in McCoy’s 5 A medium (Biological Industries, Kibbutz Belt HaEmek, Israel) supplemented with 10% fetal bovine serum (FBS; Biological Industries, Kibbutz Belt HaEmek, Israel). The oxaliplatin-resistant cell line HCT116/L-CHOP was maintained in RPMI 1640 medium supplemented with 10000 ng/ml oxaliplatin (Selleck, Selleck Chemicals, USA). HEK-293T, HT29, SW480, SW620, HCT8, Caco2, RKO and LOVO cells were maintained in RPMI 1640 (Biological Industries, Kibbutz Belt HaEmek, Israel) supplemented with 10% FBS. All cells were cultured at 37 °C in the presence of 5% CO_2_.

### Human tissue samples

Two cohorts of human CRC samples were collected for this study (Supplementary Table 2, Supporting information). Cohort 1 comprised 44 pairs of CRC tissues and corresponding adjacent normal tissues, which were used to verify MEIS1, FEN1 and ELFN1-AS1 expression via qPCR or western blot; cohort 2 comprised 106 pairs of paraffin-embedded CRC and corresponding adjacent normal tissues to verify MEIS1 and FEN1 expression with IHC. All human tissues were collected from the Xiangya Hospital of Central South University (Changsha, China). The patients were informed and signed informed consent forms.

### Animal study

#### Xenografts

Four- to five-week-old BALB/c (nu/nu) nude male mice housed in a specific pathogen-free facility at the Animal Department of Central South University were randomized into groups (*n* = 8 per group). Single-cell suspensions of 3 × 10^6^ cells were subcutaneously injected into the dorsal flanks of the mice. Tumors were measured twice a week, and the tumor volume was evaluated using the following formula: total tumor volume (mm^3^) = 0.5^2^ × length × width^2^. After 4 or 5 weeks, the mice were euthanized, and the tumors were excised for further experiments.

#### Tumor treatment

When tumors reached a volume of 100–150 mm^3^, mice were treated for 4 weeks as follows: (i) 0.1% DMSO in PBS by intraperitoneal injection twice per week (control group); (ii) 10 mg/kg oxaliplatin by intraperitoneal injection once per week (oxaliplatin treatment group); (iii) 10 nM ASOs by intratumoral injection twice per week (ASO treatment group); (iv) 200 mg/kg GSK126 by intraperitoneal injection every 2 days (GSK126 treatment group); (v) 10 mg/kg oxaliplatin once per week + 10 nM ASOs twice per week (oxaliplatin + ASO treatment group); or (vi) 10 mg/kg oxaliplatin once per week + 200 mg/kg GSK126 every 2 days (oxaliplatin + GSK126 treatment group).

### Statistical analysis

Statistical analysis was carried out using SPSS version 20.0 and GraphPad Prism Software 8.0. Data are presented as the mean ± standard error of the mean (SEM). Data were analyzed by Student’s *t*-test for comparison of two independent groups, one-way ANOVA followed by Dunnett’s multiple comparisons test for univariate comparisons, and the Pearson coefficient for the linear correlation between two different parameters. Survival curves were analyzed using the Kaplan–Meier method and the log-rank test. The statistical parameters can be found in the figure legends, and a *p* value of less than 0.05 was considered significant. All experiments were repeated a minimum of three times.

## Supplementary information

Supplementary Materials

## Data Availability

All data needed to evaluate the conclusions in the paper are present in the paper and/or the Supplementary Materials. Additional data are available from authors upon request.
