# Supplementary material for: Downregulation of MEIS1 mediated by ELFN1-AS1/EZH2/DNMT3a axis promotes tumorigenesis and oxaliplatin resistance in colorectal cancer
Source: Signal Transduct Target Ther. 2022 Mar 30;7:87. doi: 10.1038/s41392-022-00902-6 (PMC8964798; doi:10.1038/s41392-022-00902-6)
Supplement: Supplementary file 1 — Supplementary Materials [file 41392_2022_902_MOESM1_ESM.docx]

**Supplementary Materials for**

**Downregulation of MEIS1 Mediated by ELFN1-AS1/EZH2/DNMT3a Axis Promotes Tumorigenesis and Oxaliplatin Resistance in Colorectal Cancer**

**Authors**

Yimin Li^1^^†^, Yaqi Gan^1†^, Jiaxin Liu^1†^, Juanni Li^1^, Zhengwei Zhou^1^, Ruotong Tian^2^, Ruizheng Sun^3^, Jiaqi Liu^1^, Qing Xiao^1^, Yuanyuan Li^1^, Pengyan Lu^1^, Yulong Peng^1^, Yuqian Peng^2^, Guang Shu ^2^, Gang Yin^1,4^*

† These authors contributed equally to this work.

* Correspondence to gangyin@csu.edu.cn

This PDF file includes:

Materials and Methods

Supplemental Reference

Supplementary Figures 1-10

Supplementary Tables 1-2

Supplementary Materials and Methods

**Bioinformatics Analysis:**

**Data collection**

The TCGA cohort data were available at the Genomic Data Commons (GDC) website (<https://portal.gdc.cancer.gov/>). Four independent cohorts of CRC data were downloaded from the GEO database (<http://www.ncbi.nlm.nih.gov/geo/>): GSE21510^1^, GSE32323^2^, GSE9348^3^, and GSE69657^4, 5^. TCGA colon & rectum adenocarcinoma (COADREAD) DNA methylation (HumanMethylation450) data from were downloaded via the University of California Santa Cruz (UCSC) Xena website (<https://xenabrowser.net/hub/>).

**Identification of intersected differentially expressed mRNAs in TCGA CRC**

Initially, log2 (FPKM+1) transformation and quantile normalization were performed on each dataset. Differentially expressed genes (DEGs) with log fold change > 1 and P value < 0.05 were obtained using the “limma” package (<http://bioinf.wehi.edu.au/limma>) of R software (<http://www.bioconductor.org/>).

**Protein-RNA interaction predictions**

The CatRAPID algorithm (<http://s.tartaglialab.com/page/catrapid_group>)^6^ and RNA-protein interaction prediction database (RPISeq, <http://pridb.gdcb.iastate.edu/RPISeq/>)^7^ were used to predict the protein-RNA interaction potential.

**Method Details:**

**RNA interference**

The sequences of siRNA and the ELFN1-AS1 ASO were purchased from RiboBio (Guangzhou, China). Transfections of siRNAs, ASOs and plasmids were performed using Lipofectamine 3000 (PolyPlus-transfection, France). The sequences of the siRNAs and ASOs are listed in Supplementary Table 2. At 48 h after transfection, the cells were collected for further experiments.

Independent short hairpin RNAs (shRNAs) against different genes were cloned into a pLKO.1 vector. HEK293T cells were cotransfected with pLKO.1-based shRNAs, pREV, pGag and pVSVG (gifts from Mian Wu, University of Science and Technology of China, Hefei, China) at a ratio of 2:2:2:1. The virus particles were collected 48 h after transfection. CRC cells were infected with recombinant lentivirus transducing units in 1 μg/ml polybrene (Sigma‐Aldrich, St. Louis, MO), and transduced cells were selected with 1 μg/ml puromycin. The sequences of shRNAs are shown in Supplementary Table 2.

**Plasmids**

The pCDH-CMV-MCS-EF1-MEIS1 and pmCherry-C1-ELFN1-AS1 plasmids and parental pCDH and pmCherry-C1 vector plasmids were obtained from CUSABIO (Wuhan, China). The pcDNA3-DNMT1, pcDNA3-DNMT3a, and pcDNA3- DNMT3b vectors were kindly provided by Lingqiang Zhang (National Engineering Research Center for Protein Drugs, Beijing, China), and the pLKO.1-shEZH2-1, pLKO.1-shEZH2-2, and plvx-EZH2 vectors were kindly provided by Shan Gao (Suzhou Institute of Biomedical Engineering and Technology, Chinese Academy of Sciences, Suzhou, China). The human gene promoter region generated by PCR amplification from Caco2 cells was cloned into the pGL3-basic luciferase reporter plasmid (Promega, Madison, WI, USA). The primers used are listed in Supplementary Table 2.

**RNA isolation and real-time RT-qPCR**

Total RNA was isolated with TRIzol (Vazyme, Nanjing, China), and cDNA was synthesized using the GoScript Reverse Transcription System (Promega, Madison, WI, USA). qPCR was then performed with GoTaq qPCR Master Mix (Promega, Madison, WI, USA) to determine the relative RNA levels, with GAPDH employed as an internal control. The relative RNA levels were calculated using the 2^-ΔΔCt^ method. The sequences for the gene-specific primers used are listed in Supplementary Table 2.

**Western blot**

Lysates were resolved by electrophoresis and transferred to polyvinylidene difluoride membranes, and antibodies targeting the following proteins were used: MEIS1 (1:1000, SC-101850, USA), cleaved caspase-3 (1:1000, #9661S, USA), GAPDH (1:5000, 60004-1-Ig, USA), γ-H2A.X (1:1000, #7631S, USA), FEN1 (1:1000, ab109132, UK), EZH2 (1:1000, #5246S, USA), and DNMT3a (1:1000, ab2850, UK).

**Cell proliferation assays and *in vitro* IC50 assays**

Cell proliferation was assayed with a Cell Counting Kit-8 assay (CCK-8, 7Sea Biotech, Shanghai, China) as previously described ^8^. Cells (3000/well) were seeded in 96-well plates, cultured for 24 h and then treated with drugs for 48 h. Cell viability was measured, and the IC50 value was calculated using GraphPad Prism 8.0 (GraphPad Software, La Jolla, CA, USA). The effects of combination treatment were determined by using CalcuSyn 2.0 software^9^. The combination index (CI) was evaluated as follows: CI < 1, synergy; CI = 1, antagonism; CI > 1, additive.

**Colony formation assay**

For colony formation assays, 300 cells were seeded in 12-well culture dishes and allowed to grow until colonies were visible (10 days–2 weeks). Macroscopic cell colonies were fixed and stained with crystal violet, and the numbers of colonies were counted using ImageJ.

**EdU assay**

The EdU assay was performed according to a standard protocol. Cells (3000/well) were cultured in 96-well plates for 48 h, after which they were incubated for an additional 2 h in their respective media containing 50 μM EdU (RiboBio, Guangzhou, China).

**Annexin V-PE/7-AAD apoptosis assay**

For the apoptosis assay, cells were treated with oxaliplatin (5 μM) for 48 h. Adherent and nonadherent cells were harvested and stained using an Annexin V-PE/7-AAD apoptosis kit (Vazyme, Nanjing, China). The samples were then analyzed by flow cytometry.

**Luciferase reporter assay**

Assays were performed according to the manufacturer’s instructions after transfection of the indicated plasmids. The FEN1, SNAPC5, and ZWINT promoters were cloned into the pGL3-Basic vector (Promega, Madison, USA). HEK-293 cells were seeded in a 24-well plate in triplicate and allowed to settle for 24 h. The indicated plasmids were cotransfected with pRL-TK Renilla plasmid using Lipofectamine 3000 (PolyPlus-transfection, France). After 48 h, a luciferase assay was performed using a Dual-Glo luciferase assay kit (Promega, Madison, USA).

**Chromatin immunoprecipitation-qPCR (ChIP-qPCR)**

ChIP assays were performed using a ChIP kit (Santa Cruz Biotechnology, CA, USA) according to the manufacturer’s instructions. Briefly, CRC cells were crosslinked in 1% formalin for 10 min at 37°C, lysed in SDS buffer and sonicated to fragment the DNA. Eluted DNA fragments were analyzed by qPCR using the specific primers shown in Supplementary Table 2.

**IHC**

Protein expression was assessed with the EliVision two-step immunohistochemical method as previously described^10^. Antibodies targeting the following proteins were used: MEIS1 (1:200, ab19867, UK), Ki67 (1:100, 27309-1-AP, USA), and cleaved caspase (1:100, #9661S, USA). The degree of immunostaining was assessed by two independent pathologists and reported as the immunoreactive score.

**Immunofluorescence (IF)**

Cells were cultured in 48-well plates for 24 h and incubated with oxaliplatin (5 μm) (Selleck, Selleck Chemicals, USA) for 24 h, after which the cells were fixed with 4% paraformaldehyde and permeabilized with 0.1% Triton X-100 supplemented with 2% BSA for 60 min at room temperature. The cells were then incubated with anti-γH2A.X (1:1000, #7631S, CST) antibodies at 4°C overnight, followed by incubation with goat anti-rabbit Alexa Fluor 594 secondary antibody (1:300, Proteintech, SA00013-4) for 1 h at room temperature in the dark. The samples were stained with DAPI (Beyotime, Shanghai, China), and images were taken with a ZEISS LSM 880 microscope with Airyscan.

**Cytosolic and nuclear fractionation**

The indicated cells were resuspended in hypotonic buffer (25 mM Tris-HCl, pH 7.4, 1 mM MgCl_2_, 5 mM KCl) and incubated on ice for 5 min. An equal volume of hypotonic buffer containing 1% NP-40 was then added, and each sample was left on ice for another 5 min. After centrifugation at 4°C and 5000 ×g for 5 min, the supernatant was collected as the cytosolic fraction. The pellets were resuspended in nuclei extraction buffer (20 mM HEPES, pH 7.9, 400 mM NaCl, 1 mM EDTA, 1 mM EGTA, 1 mM DTT, 1 mM PMSF) and incubated at 4°C for 30 min. The nuclear fraction was collected after removing insoluble membrane debris via centrifugation at 12,000× g for 10 min. GAPDH and U6 served as controls for mRNA and noncoding RNA, respectively.

**Biotin RNA pull-down assay**

All steps of the pull-down experiment were performed in RNase-free conditions. ELFN1-AS1 or antisense ELFN1-AS1 was transcribed and labeled with Biotin RNA Labeling Mix (Sangon Biotech, China). Approximately 2 × 10^7^ cells were lysed in 1.0 ml of lysis buffer [50 mM Tris·HCl (pH 7.5), 150 mM NaCl, 2.5 mM MgCl_2_, 1 mM EDTA, 10% glycerol, 0.5% Nonidet P-40, 1 mM DTT, 1 U/μl SUPERase in TM RNase Inhibitor (20 U/μl; Ambion), Protease Inhibitor Mixture (Roche)]. Cell lysates were incubated with streptavidin beads coated with biotin-labeled RNA probes at 4°C for 4 h overnight. The beads were washed five times in RIP buffer and eluted in SDS loading buffer. The retrieved proteins were separated by SDS-PAGE and processed for immunoblotting. The primers for ELFN1-AS1 and its deletion fragments for *in vitro* transcription are provided in Table S2.

**RIP**

RIP was performed with an RNA Immunoprecipitation (RIP) Kit (Bes5101, BersinBio, China) according to the manufacturer’s instructions. Briefly, approximately 1×10^7^ cells were lysed in polysome lysis buffer supplemented with RNase inhibitor and protease inhibitor on ice 10 min before centrifugation, after which the resulting cell lysates were incubated with the indicated antibody overnight at 4°C followed by protein A/G beads at 4°C for 1 h. After extensive washes using RIP wash buffer, the bead-bound immunocomplexes were treated with proteinase K at 55°C for 1 h. RNA was extracted using TRIzol (Vazyme, Nanjing, China). Purified RNAs were then subjected to qPCR analysis as described above.

**Genomic DNA extraction and bisulfite sequencing**

Genomic DNA was isolated using a MiniBEST Universal Genomic DNA Extraction Kit Ver. 5.0 (TaKaRa, Japan) and prepared with EZ DNA Methylation-GOLDTM Kits (Zymo Research, LA, USA) according to the manufacturer’s protocol. CpG islands on the MEIS1 promoter region and primers for bisulfite sequencing PCR (BSP) were predicted via “MethPrimer” (<http://www.urogene.org/methprimer/>). PCR products were cloned into the pClone007 Simple Vector Kit (Tsingke, Beijing, China), and 5 clones were sequenced. DNA fragments were amplified using primers for bisulfite sequencing and are provided in Supplementary Table 2.

**Supplemental References**

1. Tsukamoto, S. et al. Clinical significance of osteoprotegerin expression in human colorectal cancer. *Clin. Cancer Res.* **17**, 2444-2450 (2011).

2. Khamas, A. et al. Screening for epigenetically masked genes in colorectal cancer Using 5-Aza-2'-deoxycytidine, microarray and gene expression profile. *Cancer Genomics Proteomics* **9**, 67-75 (2012).

3. Hong, Y., Downey, T., Eu, K.W., Koh, P.K. & Cheah, P.Y. A 'metastasis-prone' signature for early-stage mismatch-repair proficient sporadic colorectal cancer patients and its implications for possible therapeutics. *Clin. Exp. Metastasis* **27**, 83-90 (2010).

4. Li, S., Lu, X., Chi, P. & Pan, J. Identification of HOXB8 and KLK11 expression levels as potential biomarkers to predict the effects of FOLFOX4 chemotherapy. *Future Oncol.* **9**, 727-736 (2013).

5. Tong, M. et al. Identifying clinically relevant drug resistance genes in drug-induced resistant cancer cell lines and post-chemotherapy tissues. *Oncotarget* **6**, 41216-41227 (2015).

6. Agostini, F. et al. catRAPID omics: a web server for large-scale prediction of protein-RNA interactions. *Bioinformatics* **29**, 2928-2930 (2013).

7. Muppirala, U.K., Honavar, V.G. & Dobbs, D. Predicting RNA-Protein Interactions Using Only Sequence Information. *BMC Bioinformatics* **12**, 489 (2011).

8. Gan, Y., Li, Y., Li, T., Shu, G. & Yin, G. CCNA2 acts as a novel biomarker in regulating the growth and apoptosis of colorectal cancer. *Cancer Manag. Res.* **10**, 5113-5124 (2018).

9. Hong, J. et al. CHK1 targets spleen tyrosine kinase (L) for proteolysis in hepatocellular carcinoma. *J. Clin. Invest.* **122**, 2165-2175 (2012).

10. Li, T. et al. Methylation-mediated repression of MiR-424/503 cluster promotes proliferation and migration of ovarian cancer cells through targeting the hub gene KIF23. *Cell Cycle* **18**, 1601-1618 (2019).

Supplementary Figures

**
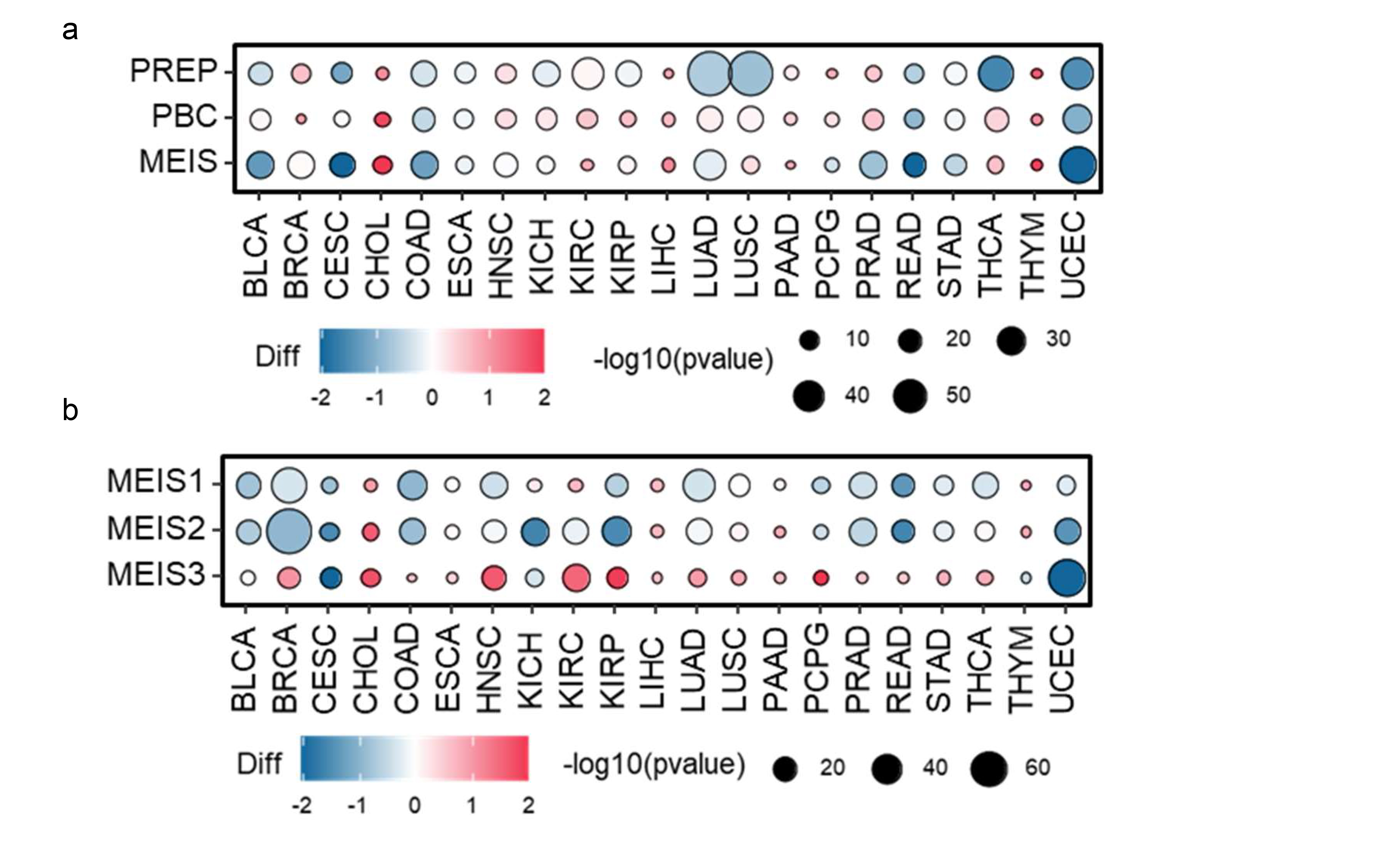
Figure S1. MEIS1 expression is downregulated in TCGA-COAD.**

(a) The TALE subfamily score among all samples grouped by cancer from the TCGA. (b) Expression levels of MEIS family genes among all samples grouped by cancer from the TCGA.


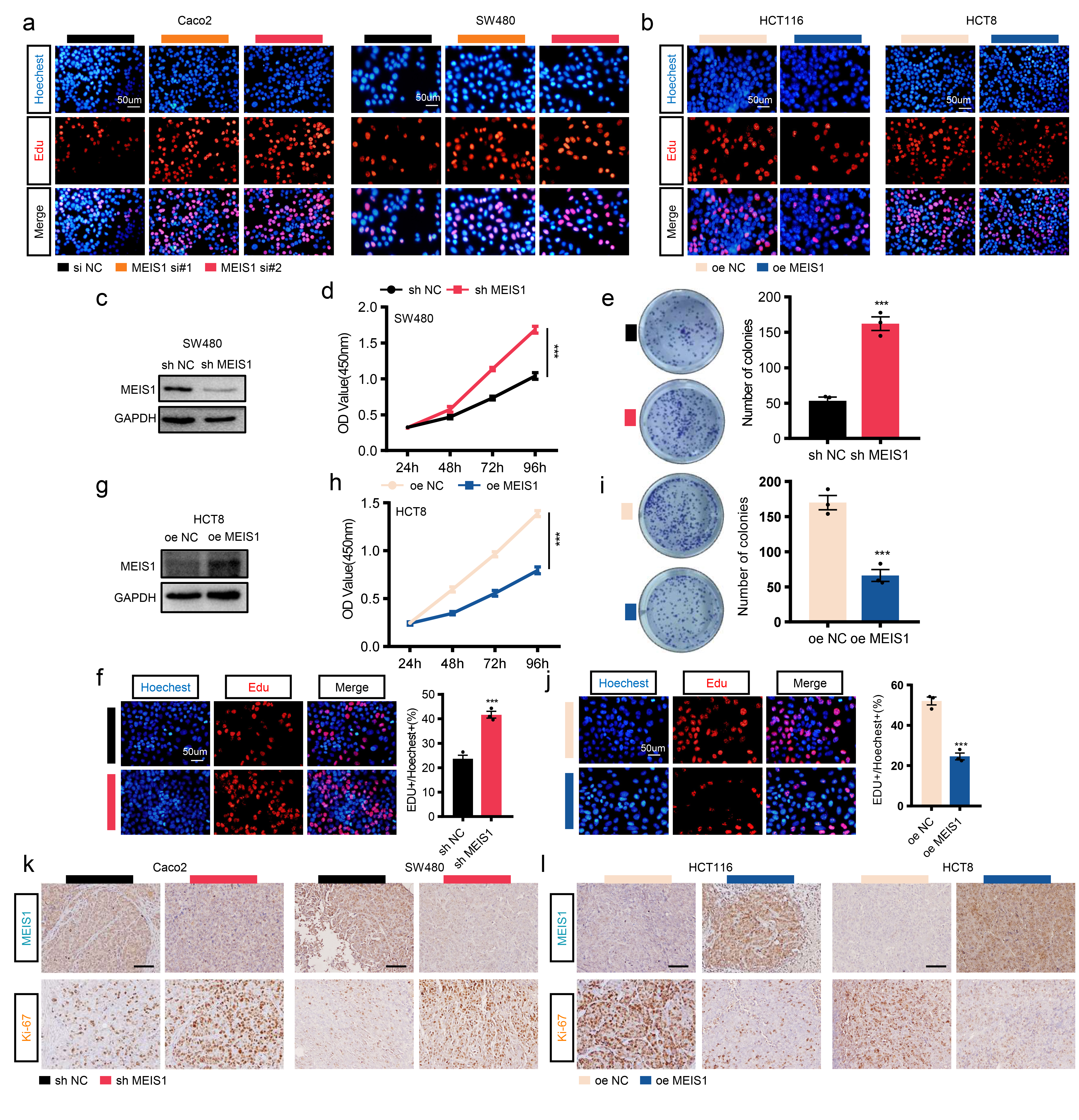


**Figure S2. MEIS1 inhibits CRC cell proliferation *in vitro* and *in vivo***

(a-b) Representative images of EdU assays of CRC cells with MEIS1 knockdown or overexpression. Scale bar: 50 μm. (c) Stable knockdown of MEIS1 in SW480 cells was confirmed by Western blot. (d-f) The proliferative ability of cells with stable MEIS1 knockdown was determined by the CCK-8 assay (d), colony formation assay (e), and EdU assay (f). (g) Stable overexpression of MEIS1 in HCT8 cells was confirmed by Western blot. (h-j) The proliferative ability of cells with stably overexpressing MEIS1 was determined by the CCK-8 assay (h), colony formation assay (i), and EdU assay (j). (k-l) Representative images of IHC of MEIS1 and Ki67 in the harvested xenografts. Scale bar, 100 µm. Data are presented as the mean ± SEM from three independent experiments. ***p<0.001.

**
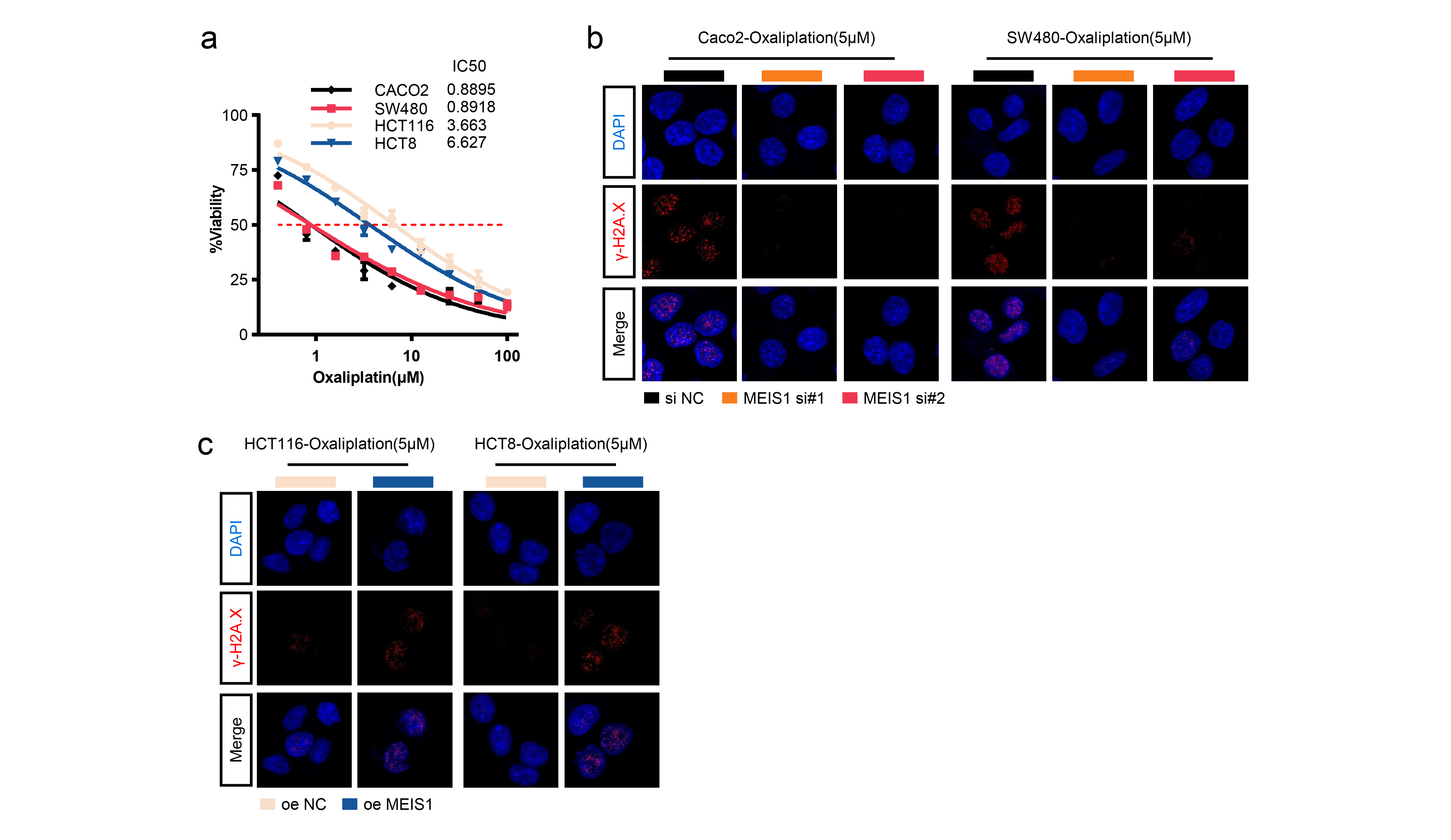
**

**Figure S3.** **MEIS1 inhibits oxaliplatin resistance and DNA damage repair in CRC cells *in vitro***

(a) CRC cells were treated with increasing concentrations of oxaliplatin for 48 h. (b-c) Representative IF images of CRC cells.

**
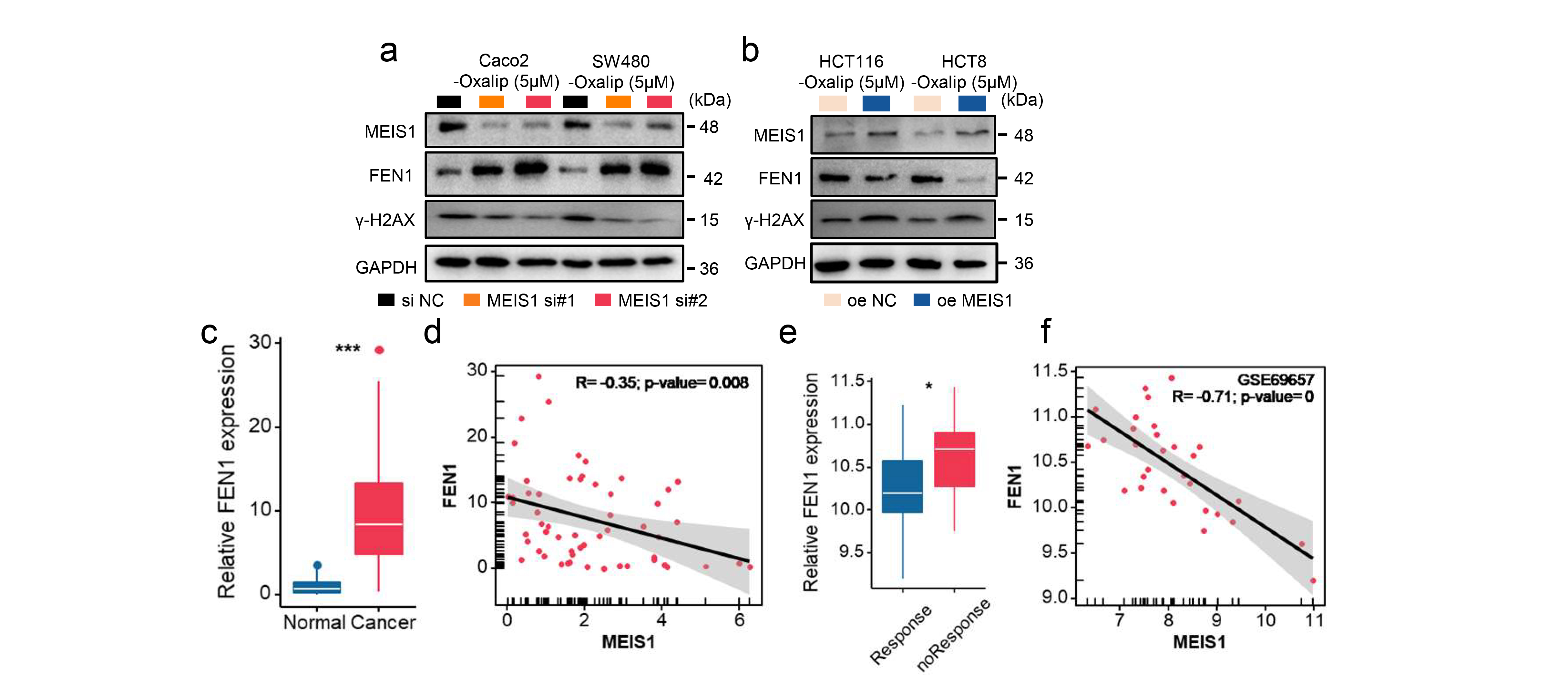
**

**Figure S4.** **MEIS1 regulates FEN1 expression at the transcriptional level, thus affecting proliferation and oxaliplatin resistance in CRC cells.**

(a-b) Western blotting was used to examine the expression levels of MEIS1, FEN1 and γ-H2A.X in CRC cells treated with oxaliplatin (5 μM) for 24 h. (c) qPCR of FEN1 expression in 12 normal samples and 44 CRC samples. (d) Correlation analysis of MEIS1 and FEN1 expression levels in 12 normal colorectal samples and 44 CRC samples. (e) FEN1 mRNA levels in the GSE69657 dataset. (f) Correlation analysis of MEIS1 and FEN1 expression levels in the GSE69657 dataset.

**
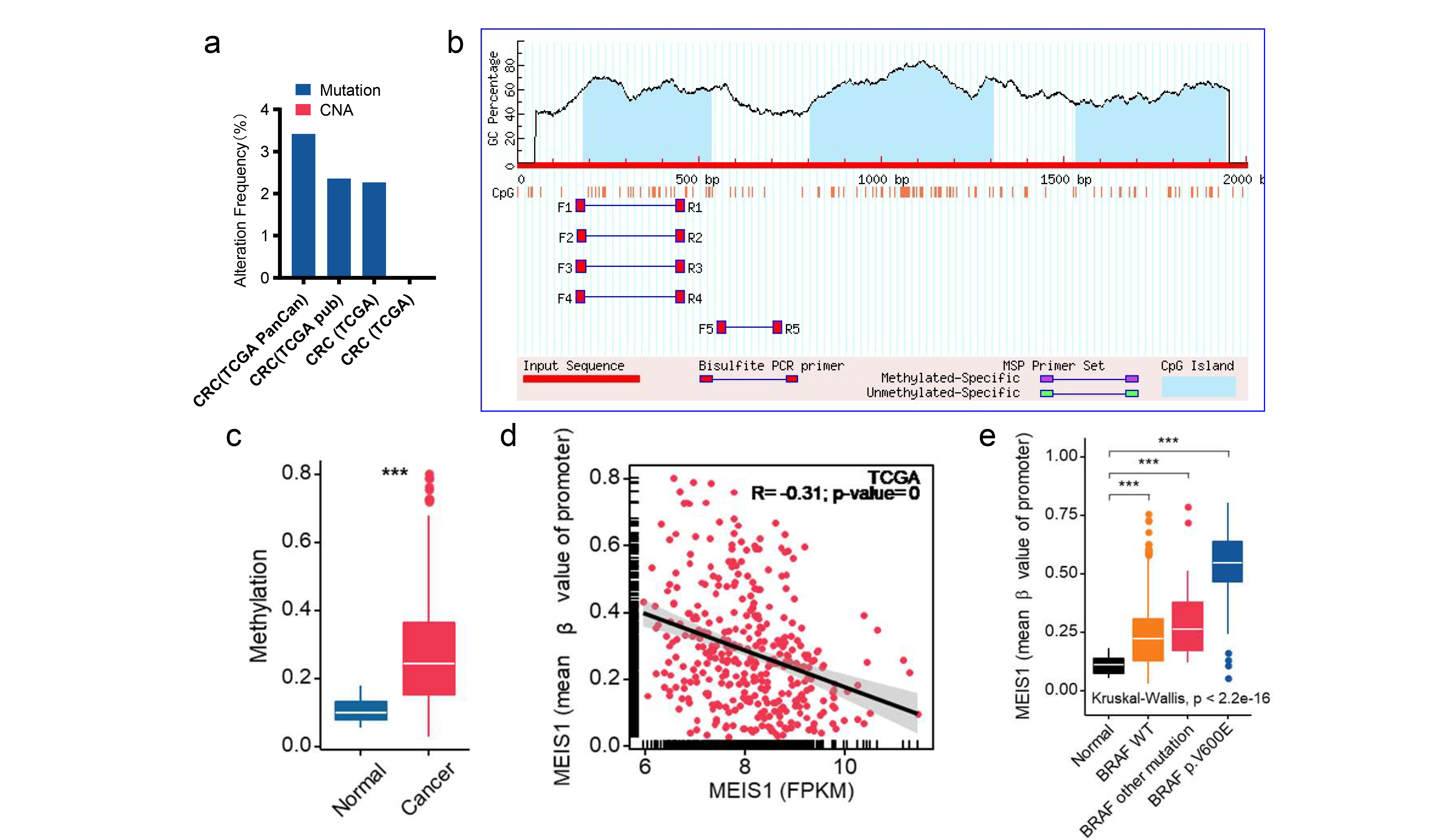
**

**Figure S5. EZH2/DNMT3a localization to the MEIS1 locus induces in the apparent silencing of MEIS1 in CRC**

(a) Copy number alterations (CNAs) and mutations of MEIS1 were analyzed by cBioPortal. (b) CpG islands in the promoter region of MEIS1 were predicted using MethPrimer software. (c)The MEIS1 DNA methylation status in CRC and normal tissues from the TCGA. The mean β values of the ten MEIS1 probes represent the DNA methylation status. (d) Correlation of MEIS1 expression with MEIS1 DNA methylation status in CRC and normal tissues as indicated by the Pearson correlation coefficient. (e) The association between BRAF mutation and MEIS1 was analyzed from TCGA dataset. ***p<0.001.

**
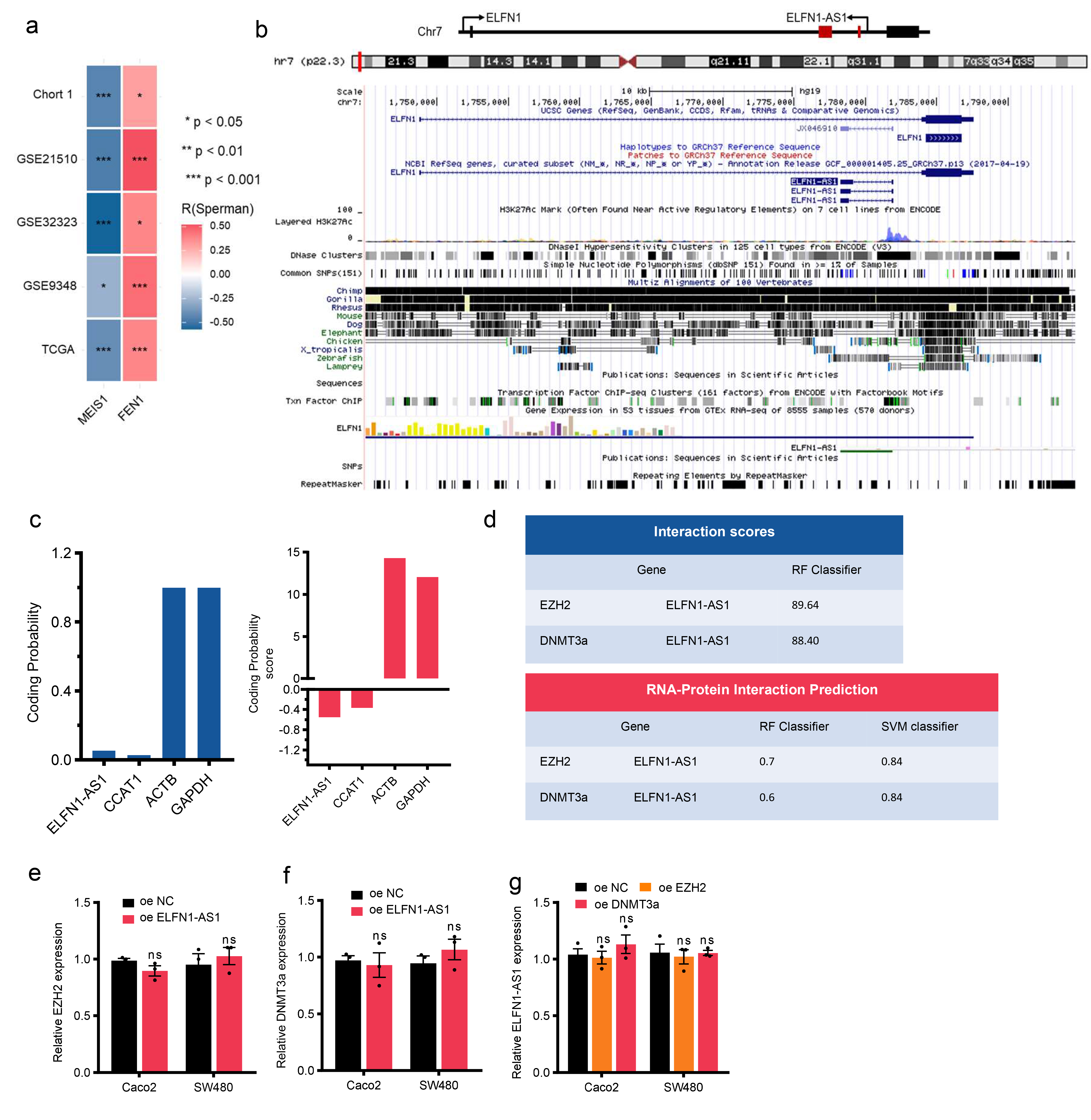
**

**Figure S6. ELFN1-AS1 recruits EZH2/DNMT3a to the MEIS1 promoter to inhibit MEIS1 expression**

(a) The correlation analysis of ELFN1-AS1 with the expression of MEIS1 or FEN1 expression in chort 1, GSE21510, GSE32323, GSE9348, and TCGA datasets by the Pearson correlation coefficient. (b) Schematic showing the ELFN1-AS1 (red) gene region of chromosome 7. ELFN1-AS1 is located at the intron of the human ELFN1 gene according to the UCSC Genome Browser (<http://genome.ucsc.edu/>). (c) The coding potential of ELFN1-AS1 was analyzed using CPAT (http://lilab.research.bcm.edu/cpat/) (left) and CPC (http://cpc.cbi.pku.edu.cn/) (right). CCAT1 served as a control noncoding RNA, whereas ACTB and GAPDH served as control coding RNAs. (d) The catRAPID algorithm (left) and RPISeq (right) results showed that EZH2 and DNMT3a were predicted to interact with ELFN1-AS1. (e-f) qPCR analyses of EZH2 and DNMT3a mRNA levels in Caco2 and SW480 cells transfected with oe-NC or oe-ELFN1-AS1. (g) qPCR analysis of ELFN1-AS1 mRNA levels in Caco2 and SW480 cells transfected with oe-NC, oe-EZH2 and oe-DNMT3a. Data are presented as the mean ± SEM from three independent experiments. ns, no significance.


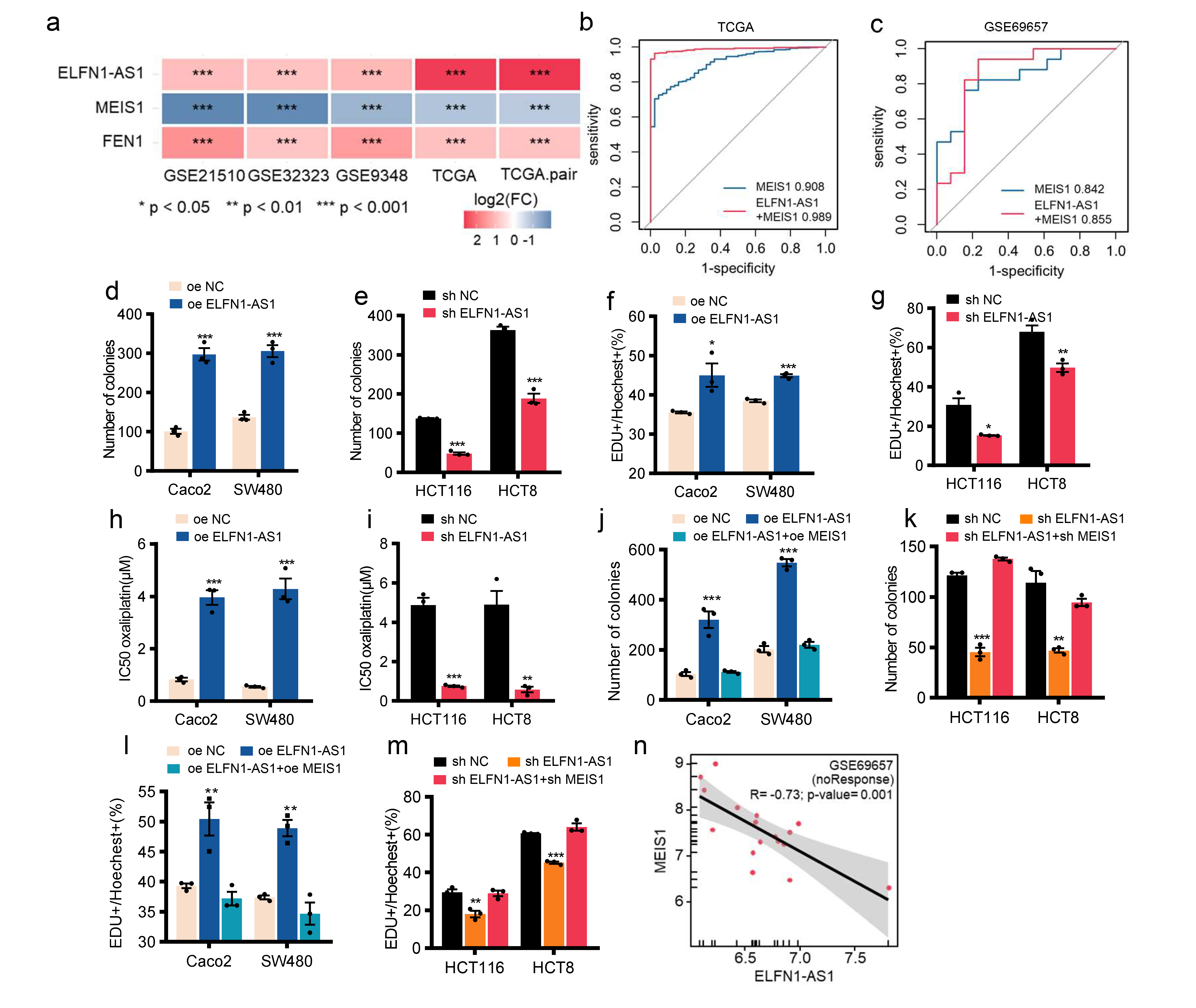


**Figure S7. ELFN1-AS1 affects cell proliferation and drug resistance by inhibiting MEIS1.**

(a) The expression of ELFN1-AS1, MEIS1 and FEN1 in CRC samples and normal colorectal samples from the GSE9348, GSE21510, GSE32323, and TCGA datasets. (b) ROC curves indicating the sensitivity and specificity of MEIS1 alone or in combination with ELFN1-AS1 for predicting the diagnosis of CRC patients from the TCGA dataset. (c) ROC curves for predicting oxaliplatin resistance in cases from the GSE69657 dataset. (d-g) CRC cell proliferation was evaluated by the colony formation (d-e) and EdU assays (f-g). (h-i) The IC50 values were calculated based on the results of the CCK8 assay. (j) Correlation analysis of ELFN1-AS1 and MEIS1 expression levels in patients from the GSE69657 dataset who did not respond to oxaliplatin. (k-n) CRC cell proliferation was evaluated by the cell colony formation (k-l) and EdU assays (m-n). Data are presented as the mean ± SEM from three independent experiments. *p < 0.05, **p < 0.01, ***p<0.001.

**
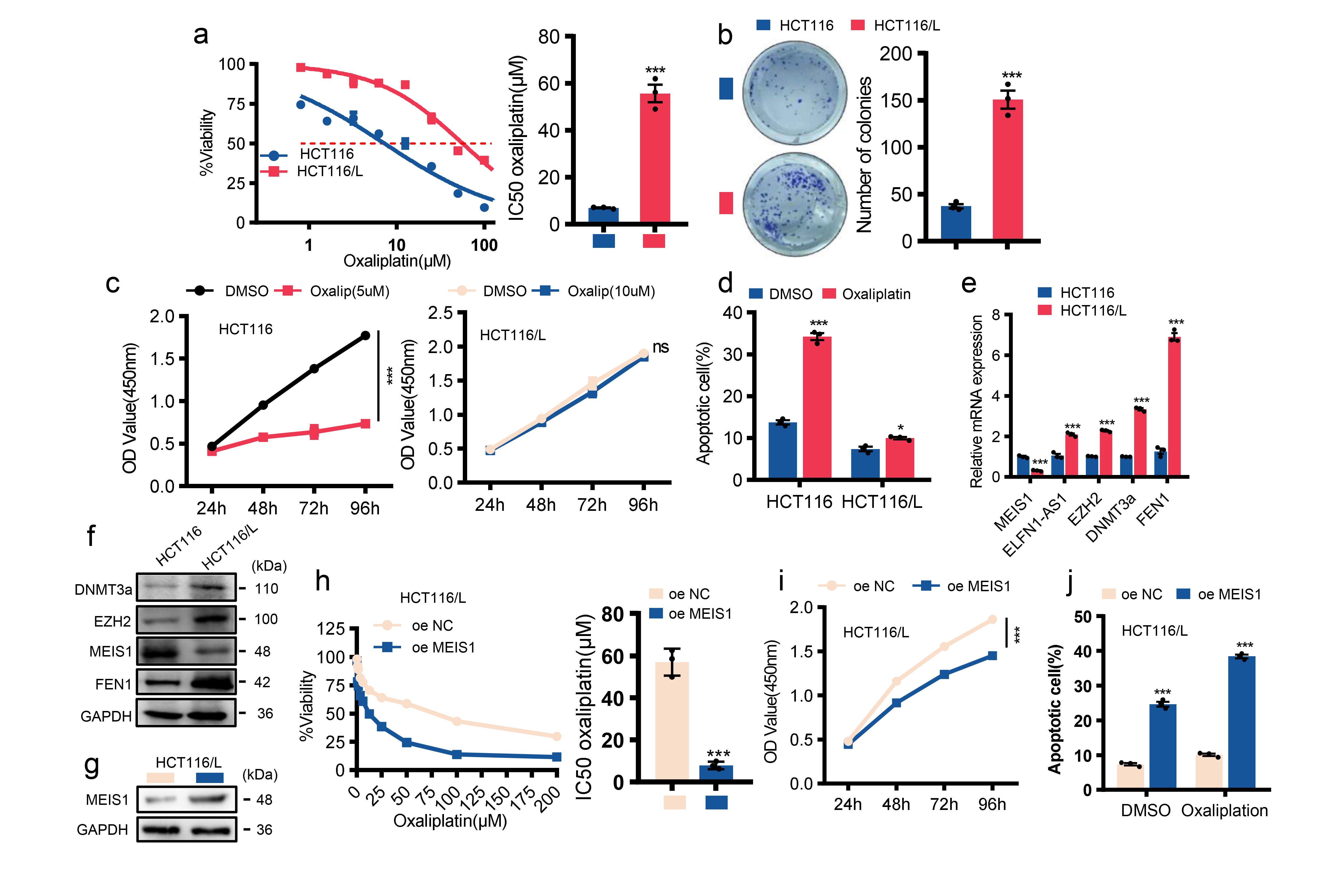
**

**Figure S8. Overexpression of MEIS1 increases the sensitivity of drug-resistant HCT116/L-OHP cells to oxaliplatin**

(a) The IC50 values of oxaliplatin in resistant HCT116 (HCT116/L-OHP) and parental HCT116 cells were detected. (b) Cell proliferation was evaluated by the colony formation assay. (c) The proliferation ability of HCT116 and HCT116/L-OHP treated with 5 µM and 10 µM oxaliplatin. (d) Flow cytometry was used to detect the apoptosis rate of HCT116 and HCT116/L-OHP cells in the presence or absence of oxaliplatin. (e-f) The mRNA and protein levels of DNMT3a, EZH2, MEIS1 and FEN1 were determined by RT-qPCR and Western blot, respectively, in HCT116/L-OHP cells and parental HCT116 cells. (g) Verification of the effect of MEIS1 overexpression on HCT116/L-OHP cells by Western blot. (h-i) Comparison of the IC50 value of oxaliplatin in oe-MEIS1 or oe-NC HCT116/L-OHP cells (h) and determination of the cell proliferative ability with a CCK-8 assay (i). (j) Flow cytometry to detect the effect of MEIS1 expression level on the apoptosis rate of HCT116/L-OHP cells.


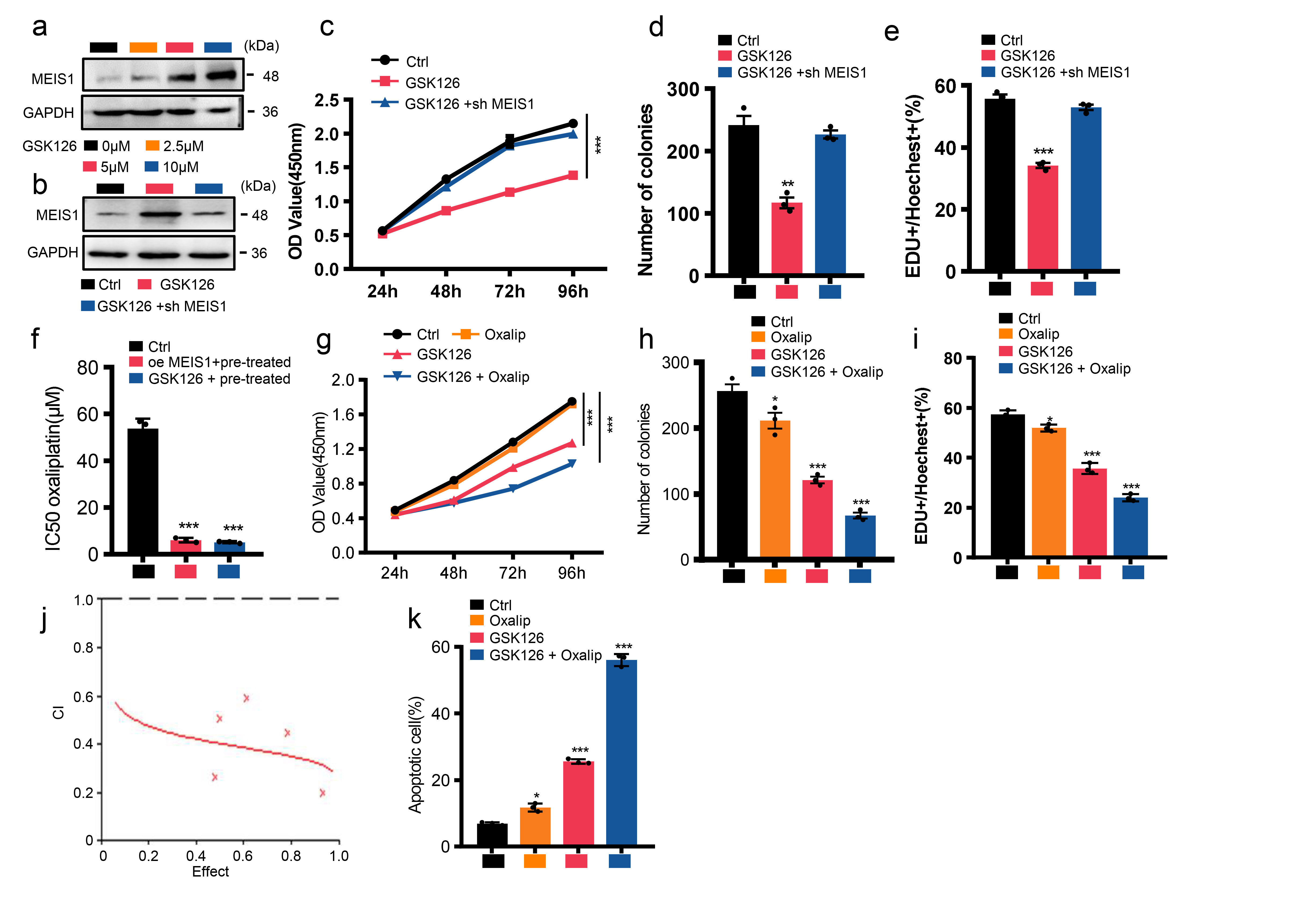


**Figure S9. The EZH2 inhibitor GSK126 reverses the resistance of CRC cells to oxaliplatin**

(a) The protein level of MEIS1 in cells treated with different concentrations of GSK126 was detected by Western blot. (b) Western blot of MEIS1 expression in HCT116/L-OHP cells treated with GSK126, GSK126+sh MEIS1 or Ctrl. (c-e) The proliferative ability of HCT116/L-OHP cells treated with GSK126, GSK126+sh-MEIS1 or Ctrl was determined by the CCK-8 (c), colony formation (d), and EdU assays (e). (f) The IC50 values of oxaliplatin in HCT116/L-OHP cells treated with oe-MEIS1+oxaliplatin (10 µM), GSK126 + oxaliplatin (10 µM) or Ctrl. (g-i) The proliferative ability of HCT116/L-OHP cells treated with oxaliplatin (10 µM), GSK126, GSK126 + oxaliplatin (10 µM), or Ctrl was determined by the CCK-8 (g), colony formation (h), and EdU assays (i). (j) The combination of oxaliplatin with GSK126 on cell proliferation is synergistic. HCT116/L-OHP cells were exposed to various concentrations of oxaliplatin and GSK126 alone or two drug-combination at a fixed ratio. Cell viability was measured by CCK8 assay.CI was calculated by using the Calcusyn 2.0 software. (k) The apoptosis level of HCT116/L-OHP cells treated with GSK126 combined with oxaliplatin was determined by flow cytometry. Data are presented as the mean ± SEM from three independent experiments. *p < 0.05, **p < 0.01, ***p<0.001.

**
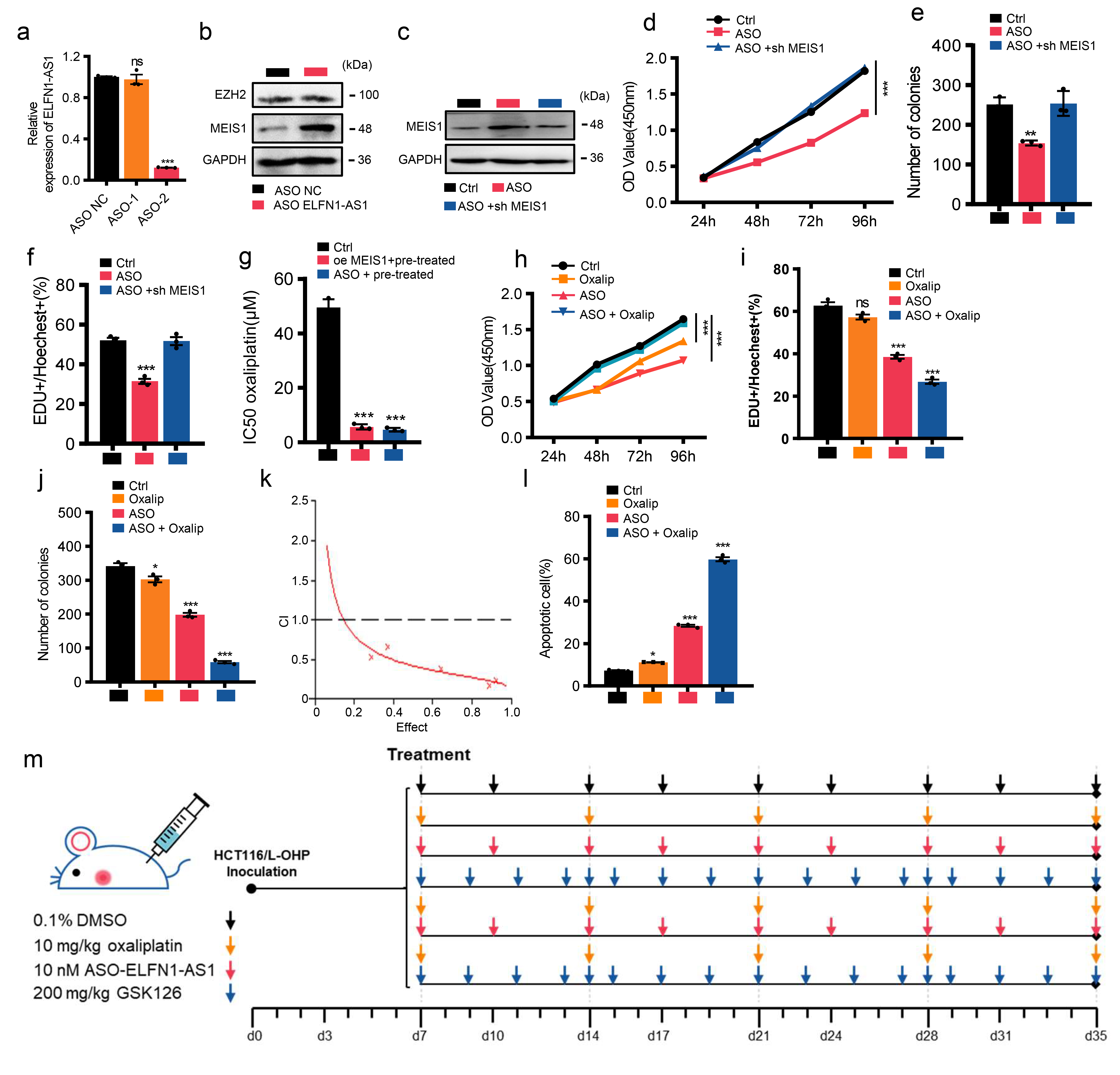
**

**Figure S10. lncRNA ELFN1-AS1 ASO restores the sensitivity of HCT116/L-OHP cells to oxaliplatin**

(a) The mRNA expression levels of ELFN1-AS1 in HCT116/L-OHP cells treated with ASO NC or ASO ELFN1-AS1 were determined by qPCR. (b) The protein levels of MEIS1 in HCT116/L-OHP cells treated with ASO NC or ASO ELFN1-AS1 were determined by Western blot. (c) Western blot of MEIS1 expression in HCT116/L-OHP cells transfected with ASO-ELFN1-AS1, ASO-ELFN1-AS1+sh-MEIS1, or Ctrl. (d-f) HCT116/L-OHP cell proliferation was determined by the CCK-8 (d), colony formation (e), and EdU assays (f). (g) The IC50 values of oxaliplatin in HCT116/L-OHP cells treated with oe-MEIS1+oxaliplatin (10 µM), ASO + oxaliplatin (10 µM) or Ctrl. (h-j) The proliferative ability of HCT116/L-OHP cells treated with oxaliplatin (10 µM), ASO ELFN1-AS1, ASO ELFN1-AS1 + oxaliplatin (10 µM), and Ctrl was determined by the CCK-8 (h), colony formation (i), and EdU assays (j). (k) The combination of oxaliplatin with ASO ELFN1-AS1 on cell proliferation is synergistic. HCT116/L-OHP cells were exposed to various concentrations of oxaliplatin and ASO ELFN1-AS1 alone or two drug-combination at a fixed ratio. Cell viability was measured by CCK8 assay.CI was calculated by using the Calcusyn 2.0 software. (l) The apoptosis level of HCT116/L-OHP cells treated with ASO ELFN1-AS1 combined with oxaliplatin as assessed by flow cytometry. (m) Schematic illustration of the treatment schedule. Data are presented as the mean ± SEM from three independent experiments. *p < 0.05, **p < 0.01, ***p<0.001, ns, no significance.

Supplementary tables

**Table S1. Clinicopathological characteristics of CRC patients**

Chort 1

| Patients characteristics |  |  | No. of patients (%) |
| --- | --- | --- | --- |
| Age(years) |  |  |  |
| <60 |  |  | 13（29.55） |
| ≥60 |  |  | 31（70.45） |
| Gender |  |  |  |
| male |  |  | 23（52.27） |
| female |  |  | 21（47.73） |
| Differentiation |  |  |  |
| well, moderate |  |  | 35（79.55） |
| poor |  |  | 9（20.45） |
| TNM stage |  |  |  |
| I~II |  |  | 19（43.18） |
| III~IV |  |  | 25（56.82） |
| T stage |  |  |  |
| T1~T2 |  |  | 16（36.36） |
| T3~T4 |  |  | 28（63.64） |
| Lymph node metastasis |  |  |  |
| NO |  |  | 19（43.18） |
| Yes |  |  | 25（56.82） |

Chort 2

| Patients characteristics |  |  | No. of patients (%) |
| --- | --- | --- | --- |
| Age(years) |  |  |  |
| <60 |  |  | 52 (49.06) |
| ≥60 |  |  | 54 (50.94) |
| Gender |  |  |  |
| male |  |  | 66 (62.26) |
| female |  |  | 40 (37.74) |
| Differentiation |  |  |  |
| well, moderate |  |  | 87 (82.08) |
| poor |  |  | 19 (17.92) |
| TNM stage |  |  |  |
| I~II |  |  | 46 (43.4) |
| III~IV |  |  | 60 (56.6) |
| T stage |  |  |  |
| T1~T2 |  |  | 21 (19.81) |
| T3~T4 |  |  | 85 (80.20) |
| Lymph node metastasis |  |  |  |
| NO |  |  | 46 (43.40) |
| Yes |  |  | 60 (56.60) |

**Table S2. The primer sequences and antibody**

The primer sequences

| Target | Type | Sequence |
| --- | --- | --- |
| Negative Control | si NC | TTCTCCGAACGTGTCACGT |
|  | sh NC | TTCTCCGAACGTGTCACGT |
| MEIS1 | qPCR probes | F: CTGTTTGAAAGGGAAAATGCC |
|  |  | R: TGGAAGGGCCTGGGGTT |
|  | siMEIS1#1 | CTATCGATTTGGTGATAGA |
|  | siMEIS1#2 | CTCTGTCAATGACGCTTTA |
|  | shRNA | GCGTGGCATCTTTCCCAAAGT |
|  | BSP-PCR probes | F: TTATTTATATTTTTTTGAGGGTGAGG |
|  |  | R: ACTACGAAAAAAATCAATTATCAAATAATCG |
|  | pCDH | F: CCGGAATTCATGGCGCAAAGGTACGACGAT |
|  |  | R: GGGGTACCCATGTAGTGCCACTGCCCCT |
| GAPDH | qPCR probes | F: GCACCGTCAAGGCTG AGAAC |
|  |  | R: TGGTGAAGACGCCAGTGGA |
| MEIS1 promoter (CHIP) | -2079-1870 | F: CTAAGGAGACTACGGACGCTCG |
|  |  | R: CTTCATCCGAAGATTGTTTGGGAAAG |
|  | -1896-1694 | F: CTTTCCCAAACAATCTTCGGATGAAG |
|  |  | R: CGAACTGCCAGTCCCTATTCC |
|  | -1715-1505 | F: GGAATAGGGACTGGCAGTTCG |
|  |  | R: GCTGAAACCTCGCAGAAAACTC |
|  | -1527-1314 | F: GAGTTTTCTGCGAGGTTTCAGC |
|  |  | R: CTCCCGCTCTAAGCTTCTCC |
|  | -1334-1133 | F: GGAGAAGCTTAGAGCGGGAG |
|  |  | R: CGGCGAAACCTCCCAGTG |
|  | -1151-872 | F: CACTGGGAGGTTTCGCCG |
|  |  | R: GAGGCTCTTTCTCCGACCC |
|  | -891-694 | F: GGGTCGGAGAAAGAGCCTC |
|  |  | R: CGTCCTGCGCGAAGTAGAG |
|  | -713-488 | F: CTCTACTTCGCGCAGGACG |
|  |  | R: GAGAAGCCAGGTTGGGAAAG |
|  | -508-283 | F: CTTTCCCAACCTGGCTTCTC |
|  |  | R: GAAATAAATTGGGAATCAACGCCGG |
|  | -308-113 | F: CCGGCGTTGATTCCCAATTTATTTC |
|  |  | R: ACTGGAGAGGCAGAGAGGC |
|  | -132-60 | F: GCCTCTCTGCCTCTCCAGT |
|  |  | R: GTCCCCGTGCGTGTGTAAAG |
|  | qPCR probes | F: GCCAAAAAGCTGCCAATCCA |
| FEN1 |  | R: GCCAATTTTCTGGCACAGGG |
|  | shRNA | GAGAATGACATCAAGAGCTACTTTG |
| FEN1 promoter (CHIP) | P1 | F: GCAGATCAATGGCCCTTATTAGC |
|  |  | R: CTGGGTACCACCAGTCAGTG |
|  | P2 | F: CACTGACTGGTGGTACCCAG |
|  |  | R: GGTGCGCACCTGTAATCCTAT |
|  | P3 | F: GATATCTGCCTGCCTCGG |
|  |  | R: TTGAACCCAGAAGGCGGAG |
|  | P4 | F: GGGATTACAGGAGTGAACCACC |
|  |  | R: ACTCCCTGCTAGGCACTAGAT |
|  | P5 | F: CATTGTCACCGGTCAGCC |
|  |  | R: TCTGACGCGCATCTAGCTC |
|  | P6 | F: CTTCCAATTACGGGCCCTCT |
|  |  | R: GCACTTCCAACTCTCAGGC |
|  | qPCR probes | F: CCATCCGCCACATTCCTACACC |
| ELFN1-AS1 |  | R: CAACTCCAGCAGCATCATTTCCAC |
|  | shRNA | GCATCTAATCCACCTGCAGAA |
|  | ASO-h-ELFN1-AS1_001 | TCTCTTGCTCAAAATAACCC |
|  | ASO-h-ELFN1-AS1_002 | CATTCACTCCGAGACGCAGC |
| ELFN1-AS1(RNA pull down) | sense | F: TAATACGACTCACTATAGGGGGGTCTGGCCAGCGGCC |
|  |  | R: TTTGTGAGAAACCACAAGCTCCCTGG |
|  | antisense | F: GGGTCTGGCCAGCGGCC |
|  |  | R: TAATACGACTCACTATAGGGTTTGTGAGAAACCACAAGCTCCC |
|  | 1~322 | F: TAATACGACTCACTATAGGGGGGTCTGGCCAGCGGCC |
|  |  | R: GCATCTAATCCACCTGCAGAAG |
|  | 1~655 | F: TAATACGACTCACTATAGGGGGGTCTGGCCAGCGGCC |
|  |  | R: GGAGGGAGCTTCCCAGGCGG |
|  | 322~1008 | F: TAATACGACTCACTATAGGGCATCTGGAATCACTCCAC |
|  |  | R: TTTGTGAGAAACCACAAGCTCCCTGG |
|  | 655~1008 | F: TAATACGACTCACTATAGGGCTGTCATTCACTCCGAGACG |
|  |  | R: TTTGTGAGAAACCACAAGCTCCCTGG |
| FEN1-2KB（luci） | pGL3-Basic | F: CTAGCTAGCATCCCACGATTTCAGCCTGG |
|  |  | R: CCCAAGCTTGGCACTTCCTTTTCCGGTTG |
| ZWINT-2kb（luci） | pGL3-Basic | F: CGGGGTACCCAATCCCCAGCCCTGGAC |
|  |  | R: CCGCTCGAGTTCCGCCTCTGTCTCCGC |
| SNAPC4-2kb（luci） | pGL3-Basic | F: CGGGGTACCCAGGAACTGTAGGCCCCTG |
|  |  | R: CCGCTCGAGCAAGCCACATGGAAACTGGC |
| PVT1 | shRNA | GGACTTGAGAACTGTCCTTAC |
| SNHG15 | shRNA | CCTTGAGTCTCATGTTCAAGC |

The antibody

| Name | Source | Catalog number |
| --- | --- | --- |
| MEIS1 | Santa Cruz | SC-101850 |
| MEIS1 | abcam | ab19867 |
| GAPDH | Proteintech | 60004-1-Ig |
| IgG | Beyotime | A7028 |
| DNMT3a | abcam | ab2850 |
| EZH2 | CST | #5246S |
| Anti-trimethyl-Histone H3 (Lys27) Antibody | Sigma-Aldrich | 07-449 |
| γ-H2AX | CST | #7631S |
| FEN1 | abcam | ab109132 |
| Anti-Mouse | Proteintech | SA00001-1 |
| Anti-Rabbit | Proteintech | SA00001-2 |
| cleved caspase3 | CST | #9661S |
| ki67 | Proteintech | 27309-1-AP |
| CoraLite594—conjugated Goat Anti-Rabbit IgG(H+L) | Proteintech | SA00013-4 |
